# Spatial heterogeneity and hydrological fluctuations drive bacterioplankton community composition in an Amazon floodplain system

**DOI:** 10.1371/journal.pone.0220695

**Published:** 2019-08-09

**Authors:** Mariana Câmara dos Reis, Inessa Lacativa Bagatini, Luciana de Oliveira Vidal, Marie-Paule Bonnet, David da Motta Marques, Hugo Sarmento

**Affiliations:** 1 Laboratory of Microbial Processes and Biodiversity, Departamento de Hidrobiologia, Universidade Federal de São Carlos, São Carlos, SP, Brazil; 2 Programa de Pós-graduação em Ecologia e Recursos Naturais, Universidade Federal de São Carlos, São Carlos, SP, Brazil; 3 Laboratório de Ficologia, Departamento de Botânica, Universidade Federal de São Carlos, São Carlos, SP, Brazil; 4 Laboratório de Ciências Ambientais, Centro de Biociências e Biotecnologia, Universidade Estadual do Norte Fluminense, Campos dos Goytacazes, RJ, Brazil; 5 UMR 228 Espace DEV, Institute of Research for Development, Montpellier, France; 6 International Joint Laboratory, LMI OCE, Institute of Research for Development /Universidade de Brasilia, Brasilia, Brazil; 7 Institute of Hydraulic Research, Universidade Federal do Rio Grande do Sul, Porto Alegre, RS, Brazil; Stazione Zoologica Anton Dohrn, ITALY

## Abstract

Amazonian floodplains form complex hydrological networks that play relevant roles in global biogeochemical cycles, and bacterial degradation of the organic matter in these systems is key for regional carbon budget. The Amazon undergoes extreme seasonal variations in water level, which produces changes in landscape and diversifies sources of organic inputs into floodplain systems. Although these changes should affect bacterioplankton community composition (BCC), little is known about which factors drive spatial and temporal patterns of bacterioplankton in these Amazonian floodplains. We used high-throughput sequencing (Illumina MiSeq) of the V3-V4 region of the 16S rRNA gene to investigate spatial and temporal patterns of BCC of two size fractions, and their correlation with environmental variables in an Amazon floodplain lake (Lago Grande do Curuai). We found a high degree of novelty in bacterioplankton, as more than half of operational taxonomic units (OTUs) could not be classified at genus level. Spatial habitat heterogeneity and the flood pulse were the main factors shaping free-living (FL) BCC. The gradient of organic matter from transition zone-lake-Amazon River was the main driver for particle-attached (PA) BCC. The BCC reflected the complexity of the system, with more variation in space than in time, although both factors were important drivers of the BCC in this Amazon floodplain system.

## Introduction

Drained by a complex hydrological network, the Amazonian rainforest is one of the largest and most biodiverse biomes in the world [1]. The Amazon River-floodplain system comprises a complex network of permanent lotic habitats and permanent lentic habitats interconnected in extensive floodplains [2]. These floodplains contain a complex mosaic of wetland habitats, including open water environments, alluvial forests and grasslands, which are collectively referred to as floodplain lakes. Altogether, these systems play key biogeochemical processes of global relevance. In the Amazon, the annual CO_2_ outgassing is estimated at 470 TgCyr^-1^ [3], which is one order of magnitude higher than fluvial export of organic carbon to the ocean [4]. An updated budget [5] indicated that the emissions from Amazon wetlands is comparable to that emitted by rivers and streams globally [6]. A large percentage of these emissions come from floodplain lakes and flooded areas, especially those with high macrophyte coverage [7]. Moreover, the Amazon comprises a huge number of endemic species of fauna and flora, but little is known about prokaryotic diversity in Amazon floodplains, which account for most of the CO_2_ emissions through organic matter degradation [8].

In addition to spatial heterogeneity, the Amazon basin has three contrasting types of water, based on optical and physical and chemical characteristics [1]: “White water”, with a high content of suspended materials and nutrients, and pH near to neutral; “Black water”, with low concentration of nutrients, high content of dissolved humic substances and acid pH (4 > pH < 7); “Clear water”, which is an intermediate type and presents pH near to neutral [1]. Another factor of great importance is the seasonality of hydrologic conditions: the “flood pulse” [2]. The flood pulse is the major driving force in tropical floodplain systems, affecting the behavior and physiology of animals and plants [2]. Hydrologic fluctuations create a region that alternates between aquatic and terrestrial states called aquatic/terrestrial transition zone (ATTZ). The ATTZ links permanent water bodies to permanent terrestrial system. Consequently water bodies receive large amounts of terrestrial organic matter [2], which has been considered a strong driver of bacterioplankton community composition (BCC) in higher latitude freshwater ecosystems [9]. This terrestrial influence also enhances the entry of surrounding microorganisms from the soil into the aquatic system [10]. Moreover, these huge fluctuations in water level modify connectivity between the main river channel and lateral systems (floodplain lakes), affecting microorganisms dispersion [8].

Changes of organic matter sources into the floodplains lakes resulting from the flood pulse [11] also contribute to spatiotemporal heterogeneity that generates highly complex systems. During the rising waters, the main source of organic matter is the Amazon River [11], while during the high and falling waters the organic matter origin is mainly from *in situ* production, which in some floodplain lakes is dominated by macrophytes and others by phytoplankton, including cyanobacteria, as the Curuai floodplain [11,12]. The aquatic vegetation is composed of different species, mostly emergent macrophytes, that cover vast areas especially in the ATTZ [13]. These macrophytes are sources of dissolved organic matter for prokaryotic activity [14]. Recent studies indicated predictable changes in BCC in temperate river systems along a river continuum [15–17]. However, the influence of the flood pulse, typical from large tropical rivers, on spatiotemporal variability of BCC is still poorly understood (but see [8]).

A first metagenome of bacterioplankton in Amazonian waters carried out in a single sample from the Solimões River, indicated that the community was more similar to lake samples than marine or soil samples [18]. The phylum *Actinobacteria* was dominant, followed by *Proteobacteria* (*Betaproteobacteria*, *Alphaproteobacteria* and *Gammaproteobacteria*, respectively) [18]. A microbiome announcement in the lower Amazon River (main channel) and river plume (ocean) reported that the most abundant genes belonged to *Actinobacteria*, *Planctomycetes*, *Betaproteobacteria*, *Verrucomicrobia*, *Nitrospirae*, and *Acidobacteria* [19]. A comparative study of four Brazilian floodplain systems (Amazon, Pantanal, Araguaia, and Paraná) showed that BCC were similar to other freshwater systems distributed across the globe at the phyla level, with a dominance of *Actinobacteria*, *Cyanobacteria*, *Proteobacteria*, *Bacteroidetes* and *Verrucomicrobia* [20]. More recently, de Melo et al. (2019) [8] reported that Phyla *Actinobacteria*, *Proteobacteria* (classes *Alpha*, *Beta*, *Gammaproteobacteria*), *Planctomycetes* and *Cyanobacteria* had the highest relative abundances, accounting for more than 75% of the BCC in lake Janauacá, near Manaus.

In aquatic ecosystems, bacterial communities can be decomposed according to their dominant strategy or “lifestyle”. Firstly, some taxa may be found preferentially associated with particles. In general, operational taxonomic unit (OTU) richness in the particle-attached (PA) fraction is higher than in free-living (FL) [16,21,22]. Particle size [23], quality and composition of suspended material [24] are among the factors that influence the colonization and richness of prokaryotes on particles. Second, only few bacterioplankton taxa are abundant, and most taxa are present in low abundance [25]. Remaining rare may be a strategy that provides some advantages, such as low encounter rates with predators or viruses [26].

Given the global relevance of biogeochemical processes carried out in the Amazon basin, and the crucial role of prokaryotes on these processes, this study addressed the question of how BCC varies across space and time in an Amazonian floodplain system. The main goal was to elucidate which environmental factors drive BCC in a complex Amazonian floodplain system, and how the BCC (abundant, rare and all OTUs) reflect spatial and temporal variations in this system. To do that, we analyzed FL and PA prokaryotic communities through Illumina MiSeq 16S rRNA amplicon sequencing from 6 locations of Lago Grande do Curuai, a typical Amazonian floodplain system. Our sampling strategy covered the environmental conditions from the ATTZ to lake open waters, in two contrasted hydrological periods. This study addresses BCC from a flood pulse perspective in an Amazonian floodplain lake system, covering variations in space and time.

## Materials and methods

### Study site and sampling procedures

This study was carried out in Lago Grande do Curuai, a floodplain lake located in the lower Amazon portion (from 56.10°W to 55.00°W, and 2.3°S to 1.9°S), in Pará State (Brazil). The Curuai floodplain is a typical Amazon complex formed by more than 30 interconnected lakes, linked to the Amazon River by 9 channels. The watershed is approximately 3660 km^2^ including open water areas. The flooded area ranges between 575 km^2^ and 2300 km^2^ with the water level ranging between 3 m and 11 m [27]. Lago Grande do Curuai is representative of lakes on the Amazon floodplain and contains a wide range of distinct habitats, such as lakes, ATTZ and *igarapés* (low order Amazonian streams or channels) with different geochemical characteristics, comprising black and white waters [28].

**Fig 1:**
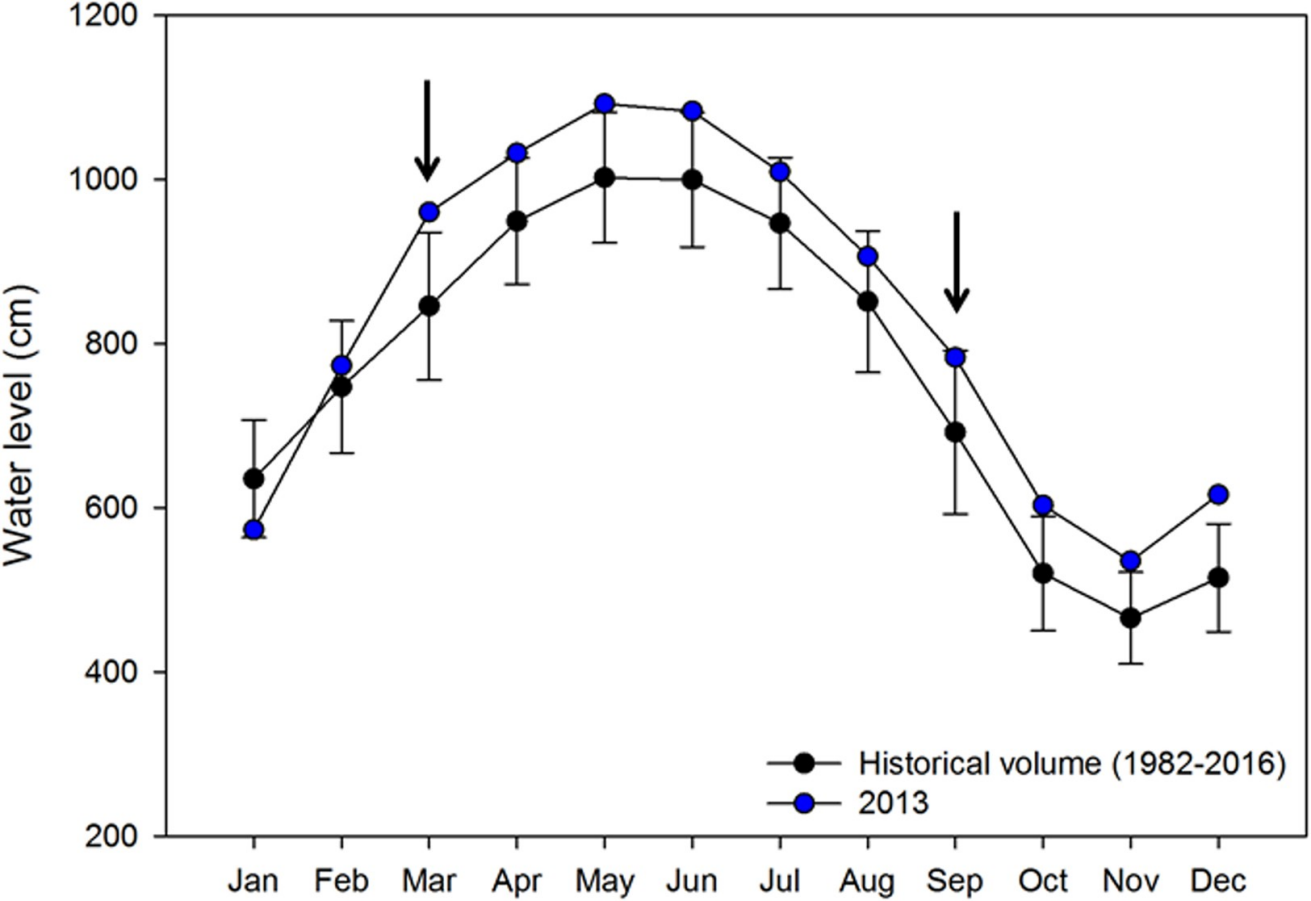
Mean water level and standard deviation in the Lago Grande do Curuai system. Black points indicate historical water level. Blue points indicate water level from the sampling year. Arrows indicate sampling dates. Data source: Agência Nacional de Águas, Hidroweb (data from 1982 to 2016).

To evaluate the spatiotemporal complexity of this system, sampling sites were selected according to their main influence: three sites located in the ATTZ (points 2,10 and 30), three sites in the open waters lake (points 15, 24 and 43), in total 6 sites. Samples were also taken in two different phases of the hydrological cycle, rising waters (March 2013), and falling waters (September 2013) (Fig 1), and in two size fractions, 3 μm and 0.2 μm (n = 24).

Water samples were collected at 1 m below the surface with Van Dorn bottle and kept in carboys pre-cleaned with 10% HCl and rinsed with Milli-Q water. In all cases, DNA filtration was performed in situ less than 6 hours after collection.

### Physical and chemical parameters

Water temperature, pH, electrical conductivity, turbidity and concentration of dissolved oxygen (DO) on the epilimnion layer were measured *in situ* using a multiparameter probe YSI EXO2 (YSI, Yellow Springs, OH, USA). Water transparency was determined with a Secchi disk. The alkalinity was determined by titrimetric methods. The concentration of total phosphorus (TP), organic phosphorus (OP) and Silica (Si) was measured with colorimetric methods [29]. For chlorophyll *a*, 250 ml of water were filtered in 0.7 pore size (Whatman GF/F glass microfiber filters) in triplicates, with low-pressure vacuum pump. Chlorophyll *a* was extracted with buffered acetone (90% acetone + 10% saturated magnesium carbonate), and the extracts were kept for 24h in the refrigerator before colorimetric determination [30]. To evaluate total suspended solids (TSS) concentration, 350 ml of lake water were filtered under moderate pressure onto acetate cellulose membranes (0.45 μm pore size) pre-dried and pre-weighted. Filters were dried for 24 h at 50°C and TSS concentration was determined gravimetrically using the dry weight of the filtered material. Concentration of total organic carbon (TOC) and dissolved organic carbon (DOC) were measured with a non-dispersive infrared method (TOC V—Shimadzu 5000). The concentration of POC was calculated by the difference of TOC and DOC. The total nitrogen (TN), total dissolved nitrogen (TDN) and nitrate (NO_3_^-^) were obtained from a non-dispersive infrared method (TOC V—Shimadzu 5000). The DOC/Chlorophyll *a* ratio was calculated by the product between DOC and Chlorophyll *a* concentration. The C:N, C:P and N:P ratios were calculated by the product of the molar concentration of total fraction for each component. The linear distance from the sampling sites to the channel (one of the main inflow source of the Amazon waters to the lake) was measured using geographic coordinates.

### DNA extraction and sequencing

Working with particle-rich Amazonian waters require some adjustments of standard methodological procedures. It is worth mentioning (might be useful for future studies), that we experienced difficulties in all steps from filtration to DNA amplification: in filtering the water (due to the high amount of particles), in DNA extraction (regular tools such as MoBio Power Soil DNA extraction kit were not efficient), in performing PCR (humic substances inhibited PCR in most samples). The problems faced in each of those steps conditioned our choices in terms of the methodologies applied.

Water samples were filtered through 3 μm polycarbonate pore size (47mm diameter) for PA fraction, and then filtered again through 1.2 μm pore size, to remove large organisms and particles. Lastly, FL prokaryotes were collected by filtration in 0.2 μm pore size (47mm diameter). Filters were kept in an ultrafreezer at -80°C until DNA extraction.

Before extraction, filters containing total DNA were submitted to enzymatic digestion with lysozyme (final concentration 1 mg ml^-1^) and Proteinase K (final concentration 0.2 mg ml^-1^). Then, total DNA was extracted using phenol-chloroform protocol followed by purification in Amicon columns (Millipore 100KDa/100.000MWCO). An additional purification step with cetyl trimethyl ammonium bromide (CTAB) 10% was realized to remove PCR inhibitory humic substances [31]. After PCR amplification, high-throughput sequencing of the V3-V4 regions of the 16S rRNA gene was performed in an Illumina MiSeq sequencing platform.

Each DNA sample was PCR-amplified in duplicated 25 μl reactions including an initial step of 95°C for 3 min, followed by 25 cycles of 98°C 20 s, 62°C for 15 s, 72°C for 15 s, and finally 72°C for 1 min, using primers 341F (5'-CCTACGGGNGGCWGCAG-3') and 805R (5'-GACTACHVGGGTATCTAATCC-3') [32]. Each reaction contained 12.5 μl of Kapa High-fidelity HOTSTART ready MIX, 0.3 μM of each primer, 10 μl of PCR-grade water, and 10 ng of DNA.

PCR products (50 μl) were purified with magnetic beads AMPURE XP kit (Bechman Coulter) and Indexed with Nextera XT kit V2 (Illumina) to separate samples. Another step of purification with magnetic beads was realized, and then the metagenomic pool was assembled with 5 μl of each library.

### Data processing and community analyses

Sequencing data were processed using UPARSE [33] in a pipeline internally implemented [34,35]. Paired-end reads were merged with PEAR [36]. Sequences were quality controlled with the following steps: all sequences shorter than 100 pb were discarded, quality dereplication checking, OTU clustering (UPARSE algorithm, similarity ≥ 97%), and filtering of chimeras (with SILVA v.119 as reference database [37]) with USEARCH [38]. Taxonomic classification was done through the BLASTn 119.1 SILVA [39] (at least 75% of similarity). All chloroplasts sequences were excluded. The SRA database of the data reported are available in the GenBank under the accession number SRP127556. The BioSample accession data are also available in GenBank under the accession numbers from SAMN08239888 to SAMN08239899. The water sampling and DNA sequencing were carried out under the permission number: A020E1F (Sistema nacional de Gestão do Patrimônio Genético e do Conhecimento Tradicional Associado, Conselho de Gestão do Patrimônio Genético, Ministério do Meio Ambiente, Brazil).

For further analyses, the OTU table was randomly subsampled (rarefied) at 11,284 (minimum number of reads) and converted into relative abundances. Abundant OTUs were that with a relative abundance over 1% within a sample, and rare OTUs were defined as having an abundance under 0.1% as previously described [26,40].

To evaluate the beta diversity among hydrological periods and influence area, we used Bray-Curtis [41] indices. Nonmetric multidimensional scaling (NMDS) ordination and cluster analyses were used to visualize this metric. We tested the homogeneity of multivariate group variances (beta dispersion) of all OTUs, abundant and rare OTUs for size fraction, hydrologic periods, and influence area using the function *betadisper* and the significance was accessed by *permutest* with 1,000 permutations. The differences among fractions, hydrologic periods and influence area for FL and PA fractions were tested using permutational multivariate analysis of variance (PERMANOVA) with Bray-Curtis index performing 1,000 permutations at sites level [42]. To identify indicative species of size fraction, hydrologic periods and influence area, we calculated the individual value index (IndVal) for each OTU using 1,000 iterations [43]. The index is calculated for each OTU as a product of their relative frequencies and relative average abundances in the determined groups. We considered as indicative species those that presented a p value < 0.05. An IndVal near 1 indicates that the specie is strongly indicative of that environment.

To determine the relative contribution of environmental variables (standardized) to the BCC patterns, we performed distance-based redundancy analysis (db-RDA) using Bray-Curtis metrics. The significance of the model and the environment variables was tested by analysis of variance test (ANOVA). Co-variance among environmental variables was tested using linear regression models and only variables that were not correlated were included. The comparison between environmental variables in the two sampling periods was done with a paired t-test. All statistical analyses were carried out in R software 3.4.3 [44] using the packages vegan [45], labdsv [46].

## Results

### Environmental parameters

A paired t-test comparison between environmental variables in the same sampling sites in the two hydrological periods revealed contrasting environmental conditions between rising and falling water periods (Table 1). Alkalinity, conductivity, TSS, POC and DOC/Chlorophyll *a* were higher during rising water and Si, pH and N:P ratio were higher during falling waters. Other parameters such as nitrogen and phosphorus concentrations had higher averages in the rising waters period but no statistically significant differences (p > 0.05).

**Table 1:** Minimum, mean and maximum values of environmental parameters sampled in both hydrological periods, rising and falling waters.

		Rising			Falling		
	Min.	Avg.	Max.	Min.	Avg.	Max.	
Temperature (°C)	30.3	**30.8**	31.7	29.8	**31.5**	34.8	
Water column depth (m)	1.8	**3.7**	5.7	2.6	**3.7**	4.3	
OD (mg/L)	4.6	**6.2**	7.5	5.9	**7.6**	11.7	
Alkalinity (mg/LCaCO_3_)	11.8	**17.4**	22.5	10.8	**13.3**	15.1	*
Conductivity (μS/cm)	38.0	**65.3**	81.0	34.0	**44.3**	59.0	*
Secchi disk (m)	0.4	**0.7**	1.7	0.4	**0.7**	0.9	
Turbidity (NTU)	4.7	**15.8**	24.4	5.0	**21.7**	48.0	
Total suspended solids (mg/L)	32.0	**54.3**	90.0	6.5	**24.7**	51.5	*
Si (mg/L)	2.0	**2.4**	3.0	2.9	**3.0**	3.3	*
pH	7.3	**7.8**	8.6	7.5	**8.3**	8.9	*
TP (ug/L)	20.0	**83.3**	150.0	30.0	**45.0**	80.0	
OP (ug/L)	10.0	**75.0**	140.0	10.0	**30.0**	50.0	
TN (ug/L)	230.0	**353.3**	430.0	190.0	**275.0**	370.0	
TDN (ug/L)	130.0	**255.0**	420.0	180.0	**253.3**	320.0	
NO_3_- (ug/L)	0.0	**46.7**	130.0	10.0	**76.7**	200.0	
TOC (mg/L)	2.6	**5.8**	8.0	3.0	**4.0**	4.9	
DOC (mg/L)	2.9	**3.8**	4.9	2.9	**3.7**	4.9	
POC (mg/L)	0.0	**2.1**	3.2	0.0	**0.2**	0.8	*
DOC/Chlorophyll *a*	0.0	**0.8**	1.8	0.1	**0.1**	0.2	*
Chlorophyll *a* (μg/L)	2.0	**38.2**	203.9	19.5	**46.2**	72.8	
C:N ratio	10.9	**19.1**	27.5	13.0	**17.3**	20.9	
C:P ratio	68.9	**227.2**	433.9	98.9	**270.9**	386.8	
N:P ratio	6.3	**12.0**	22.6	7.6	**16.1**	29.7	*
Channel distance (km)	24.2	**58.3**	90.4	24.2	**58.3**	90.4	

Asterisks indicate statistically significant differences tested by a Paired t-test (*p < 0.05).

### Bacterioplankton community composition (BCC)

**Fig 2:**
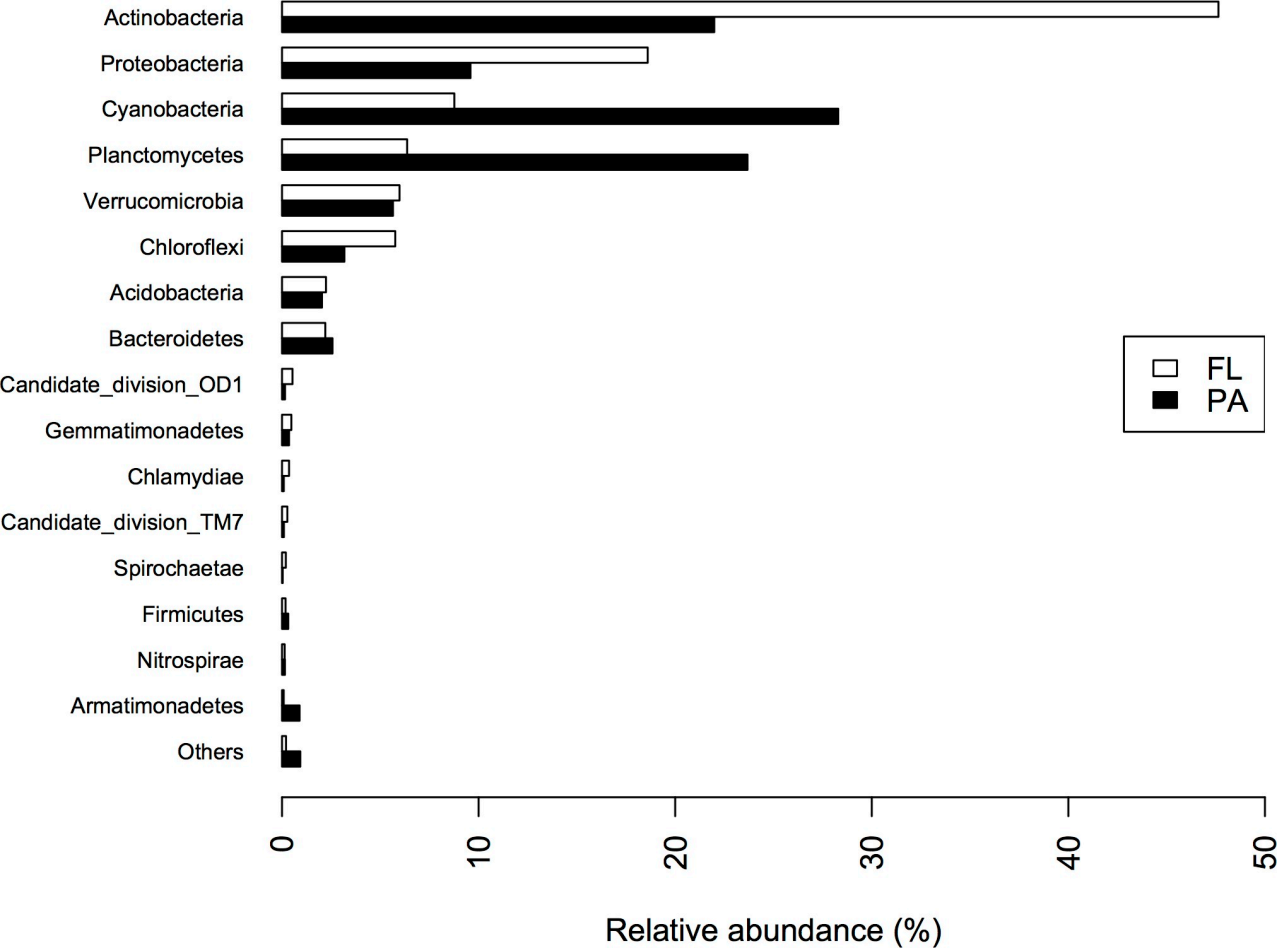
Phylum–level taxonomic composition of bacterioplankton across sampling sites (FL = free-living and PA = particle-attached).

A total of 2,519,978 high quality reads were retrieved, with a minimum of 11,284 reads per sample. Clustering at 97% of similarity resulted in a total of 2,011 OTUs, of which 1,631 remained after rarefaction. Five *Archaea* sequences were found, belonging to phylum *Thaumarchaeota*, and *Euryarchaeota*. However, these sequences were excluded in further analyses, since the primers used were not appropriate for *Archaea* diversity analyses. The most abundant phyla across all sampling sites were *Actinobacteria*, *Cyanobacteria*, *Proteobacteria* and *Planctomycetes*, in both size fractions (Fig 2). *Cyanobacteria* and *Planctomycetes* were proportionally more abundant in the larger size fraction (PA, >3μm), with mean contributions of 28.3% and 23.7% of relative abundance, respectively. *Actinobacteria* and *Proteobacteria* more abundant in the smaller size fraction (FL, <1.2 and >0.2 μm), contributing with an average of 47.6% and 18.6% of relative abundance, respectively. Interestingly, we also found a higher relative abundance of *Chloroflexi* in the FL fraction (in average 5.8%).

**Fig 3:**
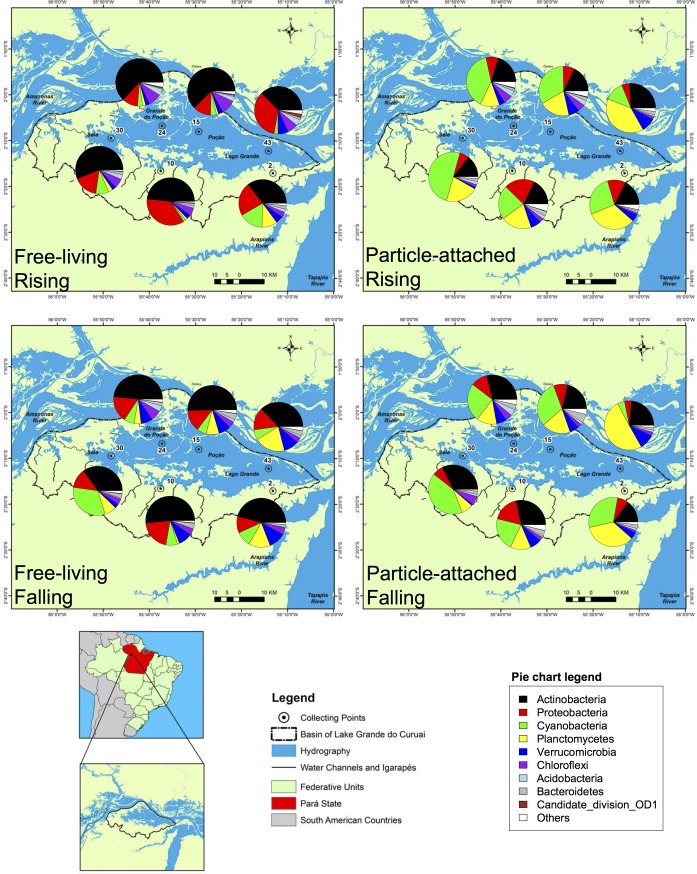
Distribution of free-living (FL) and particle-attached (PA) bacterioplankton phyla in each sampling site, in the two hydrological periods. Points 2, 10 and 30 are aquatic/terrestrial transition zone (ATTZ) influenced, 15, 24 and 43 are lake sites.

We also observed changes in the main phyla among hydrological periods and influence area (Fig 3). Considering the average of relative abundance of all sites and fractions, the most abundant phylum in rising waters was *Actinobacteria* (34.8%), followed by *Cyanobacteria* (18.4%), *Proteobacteria* (15.9%) and *Planctomycetes* (13.7%). On the other hand, during falling waters the phylum *Actinobacteria* (34.9%) was also the most abundant, followed by *Cyanobacteria* (18.7%), *Planctomycetes* (16.3%), and *Proteobacteria* (12.3%). For influence area, considering the average of relative abundance of all sites and fractions, we found that lake’s community were mainly composed by *Actinobacteria* (36.4%), followed by *Planctomycetes* (16.0%), *Cyanobacteria* (14.6%) and *Proteobacteria* (12.4%), while ATTZ sites were dominated by *Actinobacteria* (33.2%), *Cyanobacteria* (22.5%), *Proteobacteria* (15.8%) and *Planctomycetes* (14.0%).

A total of 103 OTUs were classified as abundant (contributing with > 1%), while 1607 were rare (contributing with < 0.1%). The detailed composition (at genus-level) of the four main phyla can be found in Supporting Information (S1–S4 Figs). The taxonomic distribution of abundant and rare OTUs between the main phyla were: *Actinobacteria* (in average, 26.2% in abundant and 1.1% in rare), *Cyanobacteria* (in average, 16.5% in abundant and 0.3% in rare), *Planctomycetes* (in average, 10.4% in abundant and 0.7% in rare) and *Proteobacteria* (in average, 4.0% in abundant and 3.1% in rare). Other phyla of abundant OTUs were identified as belonging to *Verrucomicrobia*, *Chloroflexi*, *Acidobacteria*, *Bacteroidetes*, *Armatimonadetes* and *Candidate_division_WS3*. The most abundant OTUs were uncultured members of the genus *Synechococcus* (*Cyanobacteria*, OTU_1), two members of *hgcI_clade* (*Actinobacteria*, OTU_4 and OTU_7), and a member of *Planctomycetaceae* (OTU_2) (S1–S4 Figs). They presented relative abundance variation among hydrological phases: *Synechococcus* (rising 13.1% and falling 10.3%), *hgcI_clade* (rising 11.3% and falling 13.4%—two OTUs) and *Planctomycetaceae* (rising 2.5% and falling 3.3%).

Some OTUs occurred only in the rare group. The main were affiliated to the phyla *Candidate_division_TM7*, *Candidate_division_OD1*, *Gemmatimonadetes*, *Chlamydiae*, *Candidate_division_WS3*, *Candidate_division_BRC1*, *BD1-5*, *Candidate_division_SR1*, *Deinococcus-Thermus*, *Firmicutes*, *SHA-109* and *Spirochaetae*. Regarding the percentage of unclassified OTUs in this work we found that 14.1% of all OTUs were not classified at class level, 59.5% at genus level and 92.4% at species level.

### Spatiotemporal patterns of BCC

We found significant values of beta dispersion for size fraction (S5 Fig) in all OTUs and abundant OTUs (F = 12.48 and p = 0.047 and F = 12.96 and p = 0.001, respectively). Since the significant beta dispersion values may compromise the interpretation of the PERMANOVA results, we performed PERMANOVA analysis for FL and PA fraction separately.

PERMANOVA analyses of FL fraction evidenced a significant effect of influence area and hydrological period for all OTUs, abundant and rare OTUs (Table 2). We did not find any significant differences for PA community among all OTUs, abundant and rare OTUs.

**Table 2:** Permutational multivariate analysis of variance (PERMANOVA) results for all OTUs, abundant and rare OTUs community composition. PERMANOVA performed using Bray-Curtis distance and 1,000 iterations.

		R^2^	p
**All OTUs**	**FL**		
	Influence area	0.24	0.002
	Hydrological period	0.16	0.022
**Abundant OTUs**	**FL**		
	Influence area	0.26	0.03
	Hydrological period	0.14	0.03
**Rare OTUs**	**FL**		
	Influence area	0.13	0.03
	Hydrological period	0.13	0.03

### Indicative OTUs of hydrologic period and influence area

We performed IndVal analyses to look for indicative OTUs of fractions, hydrologic periods and influence area. The detailed information of indicative OTUs’ taxonomy may be found in Supporting Information (S1–S3 Tables). We found 47 indicative OTUs of FL fraction and 73 of PA fraction. Among them, OTUs belonging to *Proteobacteria* phyla (mostly *Alphaproteobacteria* and *Betaproteobacteria*) were the most represented in FL fraction, followed by *Actinobacteria*, *Acidobacteria* and *Chloroflexi*. Interestingly, the most representative OTU was an *Acidobacteria* (*Subgroup_6*). For the PA fraction, most of the representatives belonged to *Proteobacteria* (mostly *Betaproteobacteria* and *Gammaproteobacteria*).

Regarding indicative OTUs of hydrologic periods, we found 103 indicative OTUs of falling waters and 72 of rising waters. Most falling waters representatives belonged to *Planctomycetes*, *Proteobacteria* (mostly *Alphaproteobacteria*) and *Cyanobacteria*. Rising waters representatives were mainly *Proteobacteria* (mostly *Betaproteobacteria*) and *Actinobacteria*.

For influence area, we found only eight indicative OTUs. ATTZ representatives were members of *Cyanobacteria*, *Planctomycetes*, *Proteobacteria* and *Actinobacteria*. Lake representatives were three OTUs belonging to *Proteobacteria* and one *Bacteroidetes* (see S1–S3 Tables for more details).

### Environmental drivers of BCC

**Fig 4:**
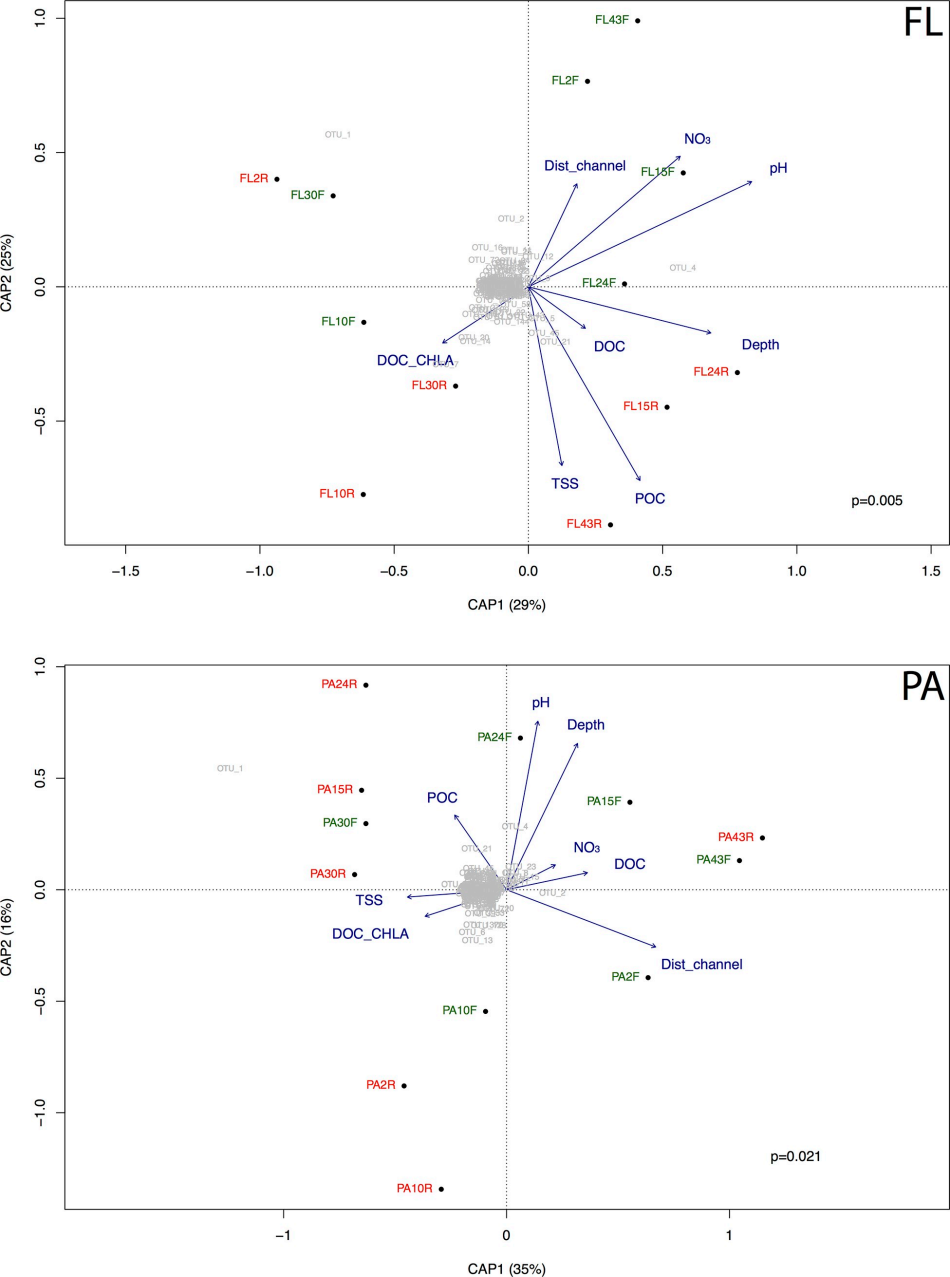
Distance-based redundancy analysis (db-RDA) of community composition using Bray-Curtis distance and standardized environmental data. Samples are identified with the size fraction, free-living (FL) and particle-attached (PA), number of the site and hydrological period, falling waters (F) in green and rising waters (R) in red. Depth, water column depth; Dist_channel, channel distance; DOC_CHLA, DOC/Chlorophyll *a* ratio; NO_3_, nitrate; POC, particulate organic carbon; TSS, total suspended solids.

In order to understand which factors explain BCC beta diversity (dissimilarity) we performed db-RDA using Bray-Curtis distance for FL and PA communities. The db-RDA model including water column depth, DOC/Chlorophyll *a* ratio, pH, POC, DOC, TSS, NO_3_- and distance from the channel explained 86% of the variation in FL fraction (eight axes, constrained proportion = 0.86) (Fig 4 FL). Most of the variation was explained by the first and second axis (CAP1 = 29% and CAP2 = 25%). The significant variables in the model were depth, pH and POC (p < 0.05). Sites were grouped by influence area (CAP1) and hydrologic periods (CAP2). Lake sites (right panel) were associated with higher pH and depth while ATTZ sites (left) were associated with lower pH and depth and higher DOC/Chlorophyll *a* ratio. The seasonality was mostly clear for rising water samples associated with higher POC and TSS concentrations while the falling waters were associated with lower values of these parameters and higher NO_3_^-^ and pH. Differences in FL fraction were mainly driven by members of genus *Synechococcus* (OTU_1) and *hgcI_clade* (OTU_4 and OTU_7).

For the PA fraction, the model explained 84% of the variation (eight axes, constrained proportion = 0.84) (Fig 4 PA). The first two axes explained most of the variation (CAP1 = 35% and CAP2 = 16%). The significant variables in the model were depth, DOC, distance from channel (p < 0.05). The PA fraction was driven by the distance from the channel and TSS concentration (upper left panel), and depth and DOC concentration (upper right panel). The community components that were associated to those variations were members of genus *Synechococcus* (OTU_1) and *hgcI_clade* (OTU_4), and family *Planctomycetaceae* (OTU_2).

## Discussion

In this study we performed a detailed analysis of BCC taking to account the spatial and temporal variations in an Amazon floodplain system. A previous metagenome study in one sample of the Solimões river found a lack of close related sequences in databases [18]. In our samples, we found a high number of OTUs that were not classified at the class level (14.1%), genus level (59.5%) and species level (92.4%). This high degree of novelty was also found in another recent work in Brazilian floodplain lakes, including Amazon samples [20]. They found that 22% of the sequences could not be identified at genus level, and 5% at class level. The Amazon biome, is known by its mega diversity of plants and animals [47]. Our results draw attention to the high degree of novelty also among bacterioplankton, which deserves further exploration.

Across the 103 OTUs with high relative abundance found in this work, the most abundant, uncultured *Synechococcus* (OTU_1), had a variable distribution among sites. Members of *Synechococcus* genus are commonly found in freshwaters and are expected to dominate and persist throughout the year in tropical regions [48,49]. *Synechococcus* belongs to smallest phytoplankton size-class (pico-phytoplankton) and has high growth rates in warm temperatures, and high surface:volume ratio that maximizes their light and nutrient uptake [50–52]. In our study, although *Synechococcus* had a higher relative abundance during the rising period (13.1%) than the falling waters (10.3%), they dominated the bacterioplankton in both hydrological periods and were one of the main representatives of community composition patterns in FL and PA communities. The uniqueness of Brazilian floodplain systems when compared with global locations has been attributed mainly to the higher abundance of *Cyanobacteria* members (Family I and Family II mostly) [20]. They assigned the higher abundance of these organisms to the ephemeral nature of floodplains lakes, since *Synechococcus* are fast growing organisms that can persist in the system and resist to the drastic changes in environmental conditions across hydrological phases.

The second and third most abundant OTUs (OTU_4 and OTU_7) were members of *hgcI_clade* (also known as acI lineage). These two OTUs presented similar relative abundance in rising (11.3%) and falling (13.4%) waters. The *hgcI_clade* is typically found in freshwaters such as reservoirs [53], estuaries [54] and lakes [55,56]. The metagenome from a Solimões sample revealed a high abundance of this group, corresponding to 73% of *Actinobacteria* sequences [18]. In our study, OTU_4 was the main representative of FL lake communities (Fig 4 FL), being associated with higher pH and deep sites. Because of their abundance and high metabolic activity, this group has an important role in the carbon cycle, and has been considered as a carbon sink since they are capable to escape predators because of their small size [57]. A recent single cell genomic study demonstrated that members of this lineage has a streamlined genome with higher gene content related to the degradation of carbohydrate and organic nitrogen compounds than other typical freshwater bacteria (*Polinucleobacter* and *LD12)* [56]. Members of the *hgcI_clade* are also capable to supplement their heterotrophic lifestyle with an anaplerotic carbon fixation metabolism, which is an alternative pathway to photoheterotrophic carbon fixation, thanks to machinery associated with actinorhodopsin genes. Altogether these characteristics explain the success of these organisms in both seasons in this Amazon floodplain system.

Another abundant OTU (OTU_2) was an uncultured member of the family *Planctomycetaceae*, which had similar relative abundance in rising (2.5%) and falling period (3.3%). *Planctomycetes* are normally found in low abundance in freshwater systems [54,55]. However, some studies reported high abundance associated with cyanobacterial blooms [58,59]. Interestingly, the relative abundance of *Cyanobacteria* members was also similar between both hydrologic periods (rising– 18.4% and falling– 18.7%).

In our study we found significant differences of beta dispersion between FL and PA communities, with higher values in the PA fraction, which can be interpreted as higher beta diversity. Recent studies in Amazon systems did not found any significant difference between FL and PA community [8,19,60]. The choice of the pore size to separate FL and PA fractions, given the nature (and size) of the particles present in Amazonian white waters has been pointed out as the main explanation for those results [8]. That study showed that 3 μm pore size might be too large to separate PA from FL communities in the Amazon [8]. In our work, we also used 3 μm for PA community, but we used a pre-filter of 1.2 μm before filter the FL community (0.22 μm). This pre-filtration is surely the main explanation to the significant differences that we found between FL and PA communities since we can have lost some cells in the 1.2 μm filter.

Still, we could observe some interesting patterns between FL and PA fractions in agreement with the results of indicator taxa. The phyla *Actinobacteria* and *Proteobacteria* were the most abundant in FL fraction and we also found a higher abundance of *Chloroflexi* in the FL fraction. Consistently, the main indicator taxa in the FL fraction were members of *Proteobacteria* (mainly *Alphaproteobacteria*) and *Actinobacteria*, and we also found four indicative OTUs belonging to *Chloroflexi*. For the PA fraction, *Cyanobacteria* and *Planctomycetes* were the most abundant, and the main indicative OTUs were *Proteobacteria* (mainly *Betaproteobacteria*) and *Bacteroidetes*, but we also found *Cyanobacteria* and *Planctomycetes* as important indicative OTUs. Although *Proteobacteria* members are usually recovered in both size fractions, the class *Betaproteobacteria* is often more abundant in the PA fraction [55]. Members of *Bacteroidetes* phyla also may compose a high proportion of PA bacteria [55]. *Planctomycetes* members can degrade phytoplankton-derived carbohydrates and are found in association with algal blooms, including cyanobacterial blooms [55,58,59]. They are also abundant in sediments from white and black waters of Amazon [61], which may explain why they are indicative of the PA fraction. A recent study in 11 north–temperate freshwater systems, also found that *Actinobacteria* was overrepresented in the FL fraction and *Cyanobacteria* and *Planctomycetes* in PA fraction [62]. They attribute it mainly to the cell size. *Planctomycetes* usually have larger cell sizes due to the presence of cellular structures and budding cell division and *Cyanobacteria* presents filamentous forms and cells that forms microcolonies, such as *Synechococcus* [62]. As abovementioned *Actinobacteria* members are known by their small size, being mentioned as ultramicrobacteria and for this reason they are more recovered in the FL fraction [57].

The significant differences between rising and falling waters were consistent with the distribution of the main phyla and the temporal indicative OTUs. We found that the main difference between both hydrological periods was in the abundance of *Proteobacteria* (rising– 15.9% and falling– 12.3%) and *Planctomycetes* (rising– 13.7% and falling– 16.3%). Regarding the indicative OTUs, the main indicators of rising waters were *Proteobacteria* (mainly *Betaproteobacteria*), *Actinobacteria* and *Chlamydiae*. For the falling waters the main indicative OTUs were *Planctomycetes*, *Proteobacteria* (mainly *Alphaproteobacteria*) and *Cyanobacteria*. Another study in the Amazon River channel found similar patterns of indicative OTUs [60]. They also found a higher proportion of *Betaproteobacteria* during the high discharge period (rising waters) and higher proportions of *Actinobacteria*, *Cyanobacteria* and *Alphaproteobacteria* in the low discharge period (falling waters) [60]. Changes between *Betaproteobacteria* and *Alphaproteobacteria* were mainly attributed to the characteristics of the system between rising and falling waters. The authors suggest that *Betaproteobacteria* organisms were favored by the dynamic conditions associated with high rainfall and high river discharge [60]. These results are also consistent with a study in Hunter river that evaluated the effects of a high flooding event on the BCC [63]. They also found that the importance of *Proteobacteria* (mainly composed by *Betaproteobacteria*) decreased from flooding conditions to low inflow conditions, when the contribution of *Alphaproteobacteria* and *Gammaproteobacteria* increased. They attributed this result to the change between high allochthonous input in high inflow period and more autochthony in low inflow period [63].

As most Amazonian floodplain system, the Curuai has a marked seasonality driven by the Amazon River flooding [64]. Usually water inflow into the floodplain starts between November and December and lasts until June [27]. From June to November/December the floodplain exports more water into the river than it receives. During the rising phase the river water enters the system and carry high amounts of sediment particles (peak between January and March) and the main source of organic matter in the floodplain is the Amazon River [11,64]. In the falling phase, water and sediments flow out of the system (peak between July and October) carrying an important pool of labile dissolved and particulate organic matter [11,64]. We found a similar pattern in the period studied. The rising waters phase was marked by significant higher values of conductivity, TSS and POC that indicates the high amount of particles in this period, and higher DOC/Chlorophyll *a* ratio, which corroborates that the main source of organic matter in this phase was from allochthonous sources. On the other hand, the falling waters phase was marked by higher N:P ratio, higher pH and low DOC/Chlorophyll *a* ratio, which is an indicator of an increase in phytoplankton contribution to DOC in this period (Table 1).

The spatial and temporal heterogeneity of Amazon conditions were reflected in the BCC. For FL fraction, the influence of the hydrological cycle was more evident in lake sites. Rising waters sites were associated with higher TSS and POC concentrations while falling water sites were associated with higher NO_3_- concentration and pH. On the other hand, ATTZ sites did not present a clear seasonality. They are more influenced by the spatial heterogeneity within the lake (distance from channel), which is associated with different degrees of allochthony of the dissolved organic matter (higher DOC/Chlorophyll *a* ratio). These results corroborate the PERMANOVA for FL fraction (all OTUs) that pointed to an effect of influence area and hydrological cycle on BCC.

The PA fraction was more influenced by the spatial heterogeneity, represented by the distance to the channel. Sites located closer to the inflow channel of the Amazon River waters to the lake, were associated with higher DOC/Chlorophyll *a* ratio, POC and TSS concentrations. While the site 43, which is the further away from these points, was associated with lower values for these variables and higher pH and depth. Again, the ATTZ sites reflected the spatial heterogeneity within the lake driven by the distance from the channel. This relationship can be associated with the characteristics of the particulate organic matter across the gradient ATTZ-lake-Amazon River. In the ATTZ there is a strong influence of terrestrial and macrophyte-derived organic matter (low DOC/Chlorophyll *a* ratio), while in the open lake waters the contribution of phytoplankton should be higher [11]. As the distance to the Amazon River decreases, there is a change to an organic matter with low algal contribution (higher DOC/Chlorophyll *a* ratio) [11]. These results are in agreement with PERMANOVA results for PA fraction, which did not detect compositional patterns among influence area and hydrological periods since the PA community was more influenced by the spatial heterogeneity within the lake. Altogether, these results show that the heterogeneity of habitats observed in Amazon systems can affect BCC differentially between size fractions.

We conclude that the BCC varies more in space than in time in this complex amazon floodplain system, but both influence area and hydrologic period are important drivers of BCC. The seasonality of BCC was clear in the FL fraction of lake samples while ATTZ sites were more influenced by the spatial heterogeneity. The PA fraction was influenced mainly by the gradient ATTZ-lake-Amazon River, reflecting changes the nature of particles in this gradient. The spatial and temporal complexity of this system was reflected in BCC. Finally, Amazonian floodplains contain a high degree of novelty in bacterioplankton, as more than half of OTUs could not be classified at genus level, opening new opportunities for further exploration of microbial biodiversity in this remote region.

## Supporting information

S1 Fig Detailed composition of phylum *Actinobacteria*. (PDF)

S2 Fig Detailed composition of phylum *Cyanobacteria*. (PDF)

S3 Fig Detailed composition of phylum *Planctomycetes*. (PDF)

S4 Fig Detailed composition of phylum *Proteobacteria*. (PDF)

S5 Fig Boxplot of beta dispersion of BCC (all OTUs and abundant OTUs) for size fractions based in dissimilarity index with Bray-Curtis distance (p = 0.047 and p = 0.001, respectively). (PDF)

S1 Table Indicator value, p value, frequency (number of times that the OTU was present among samples) and detailed taxonomy of fractions indicative OTUs. (DOCX)

S2 Table Indicator value, p value, frequency (number of times that the OTU was present among samples) and detailed taxonomy of hydrologic periods indicative OTUs. (DOCX)

S3 Table Indicator value, p value, frequency (number of times that the OTU was present among samples) and detailed taxonomy of influence area indicative OTUs. (DOCX)
