# Correction: Spatial heterogeneity and hydrological fluctuations drive bacterioplankton community composition in an Amazon floodplain system

**DOI:** 10.1371/journal.pone.0228721

**Published:** 2020-01-30

**Authors:** Mariana Câmara dos Reis, Inessa Lacativa Bagatini, Luciana de Oliveira Vidal, Marie-Paule Bonnet, David da Motta Marques, Hugo Sarmento

In the Funding section, the grant number from the funder Fundacção de Amparo à Pesquisa do Estado de São Paulo is listed incorrectly. The correct grant number is: (2014/14139-3).
