# Supplementary figures and images for: Spatial heterogeneity and hydrological fluctuations drive bacterioplankton community composition in an Amazon floodplain system
Source: PLoS One. 2019 Aug 9;14(8):e0220695. doi: 10.1371/journal.pone.0220695 (PMC6688838; doi:10.1371/journal.pone.0220695)

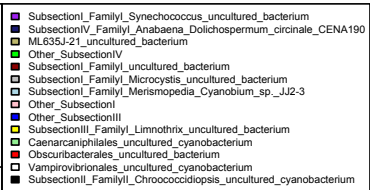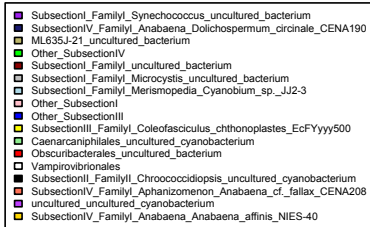

Supplement: S2 Fig — (PDF) [file pone.0220695.s002.pdf]

Relative abundance (%)

40  
30  
20  
10  
0

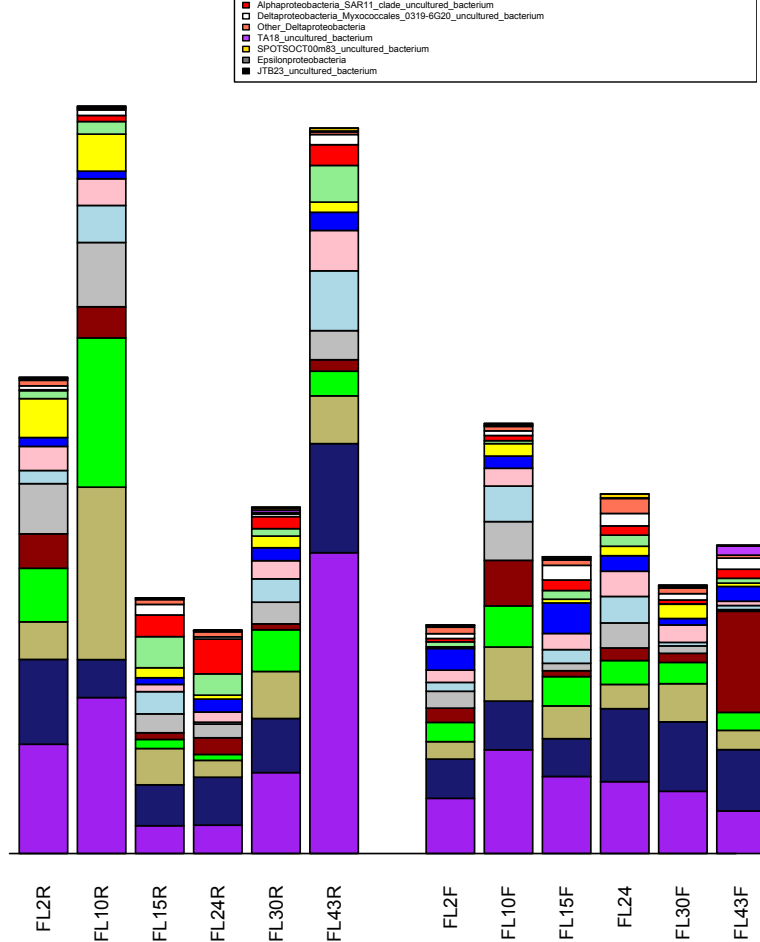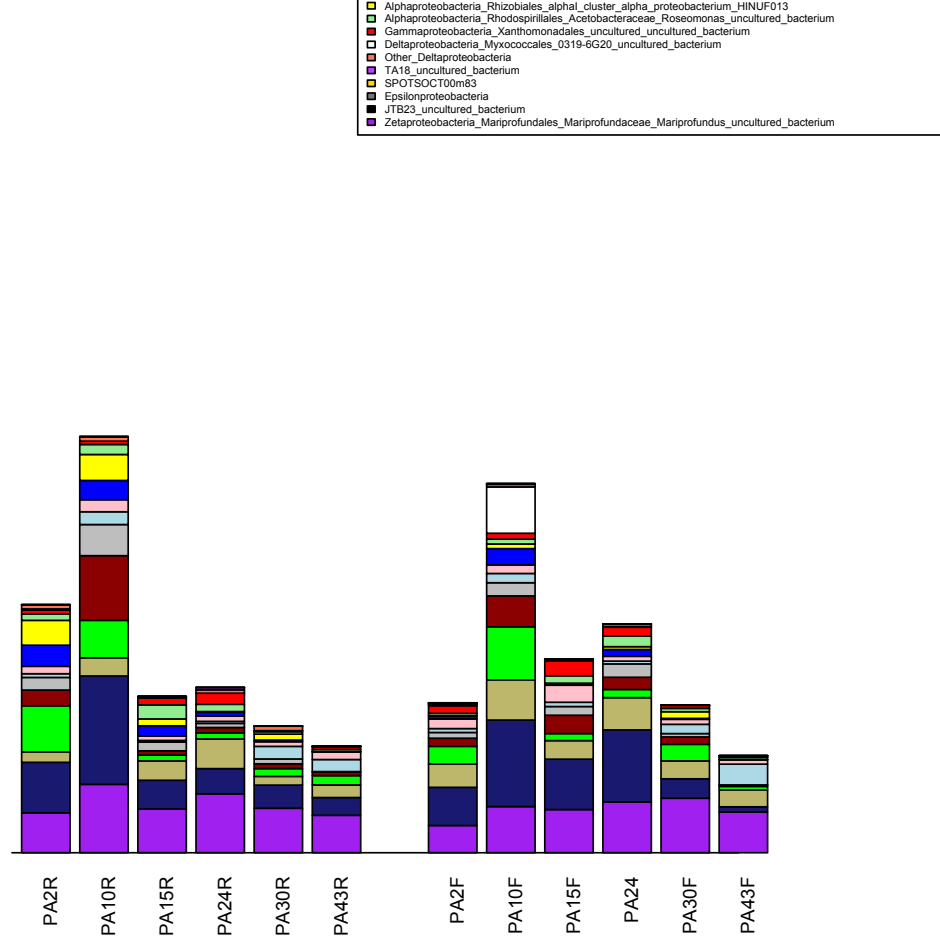

Supplement: S4 Fig — (PDF) [file pone.0220695.s004.pdf]

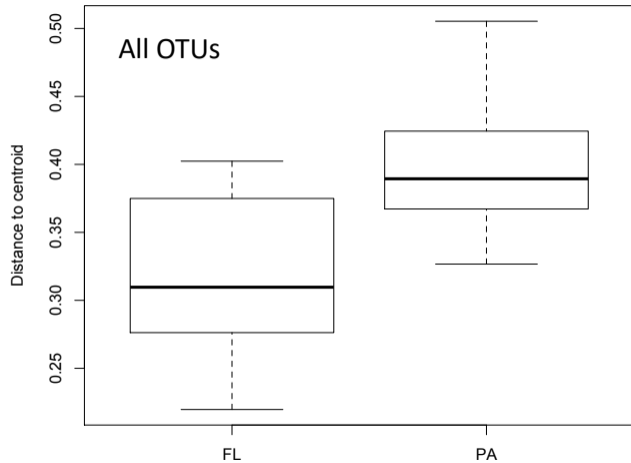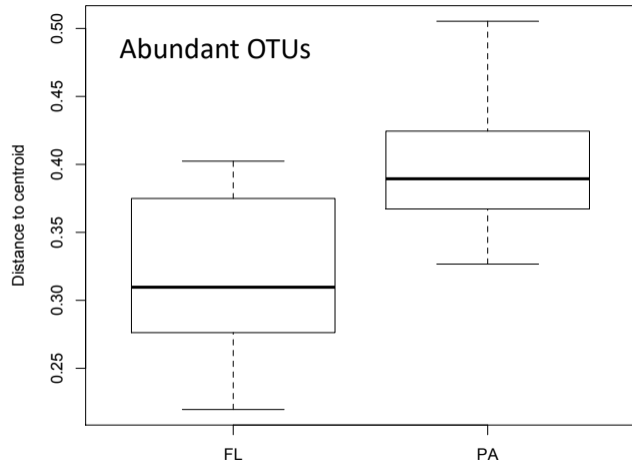

Supplement: S5 Fig — (PDF) [file pone.0220695.s005.pdf]
