# Supplementary material for: Spatial heterogeneity and hydrological fluctuations drive bacterioplankton community composition in an Amazon floodplain system
Source: PLoS One. 2019 Aug 9;14(8):e0220695. doi: 10.1371/journal.pone.0220695 (PMC6688838; doi:10.1371/journal.pone.0220695)
Supplement: S3 Fig — (PDF) [file pone.0220695.s003.pdf]

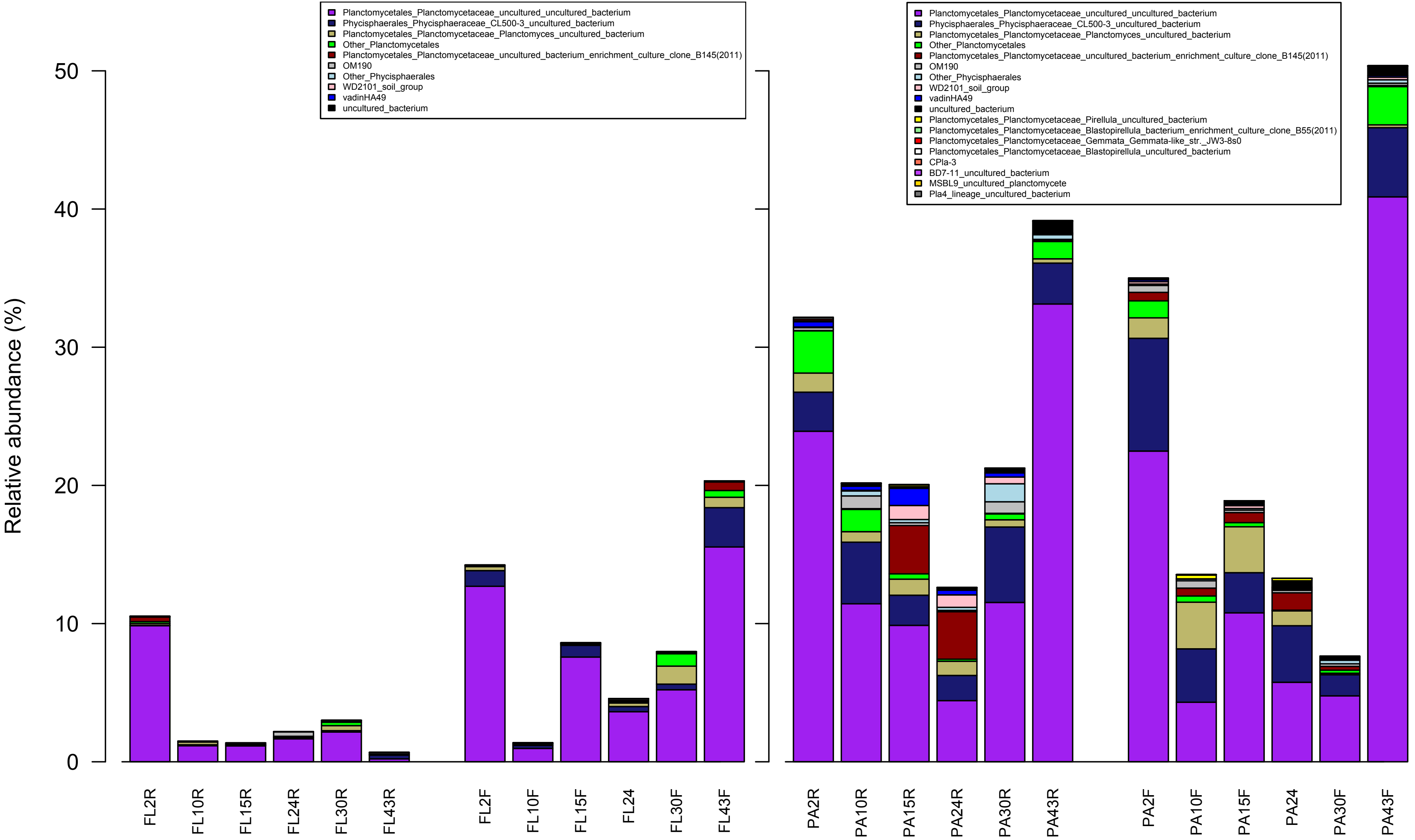

Planctomycetales_Planctomycetaceae_uncultured_uncultured_bacterium
Phycisphaerales_Phycisphaeraceae_CL500-3_uncultured_bacterium
Planctomycetales_Planctomycetaceae_Planctomyces_uncultured_bacterium
Other_Planctomycetales
Planctomycetales_Planctomycetaceae_uncultured_bacterium_enrichment_culture_clone_B145(2011)
OM190
Other_Phycisphaerales
WD2101_soil_group
vadinHA49
uncultured_bacterium
Planctomycetales_Planctomycetaceae_Pirellula_uncultured_bacterium
Planctomycetales_Planctomycetaceae_Blastopirellula_bacterium_enrichment_culture_clone_B55(2011)
Planctomycetales_Planctomycetaceae_Gemmata_Gemmata-like_str._JW3-8s0
Planctomycetales_Planctomycetaceae_Blastopirellula_uncultured_bacterium
CPla-3
BD7-11_uncultured_bacterium
MSBL9_uncultured_planctomycete
Pla4_lineage_uncultured_bacterium
Relative abundance (%)
0
10
20
30
40
50
FL2R
FL10R
FL15R
FL24R
FL30R
FL43R
FL2F
FL10F
FL15F
FL24
FL30F
FL43F
PA2R
PA10R
PA15R
PA24R
PA30R
PA43R
PA2F
PA10F
PA15F
PA24
PA30F
PA43F
