# Supplementary material for: Spatial heterogeneity and hydrological fluctuations drive bacterioplankton community composition in an Amazon floodplain system
Source: PLoS One. 2019 Aug 9;14(8):e0220695. doi: 10.1371/journal.pone.0220695 (PMC6688838; doi:10.1371/journal.pone.0220695)
Supplement: S1 Table — (DOCX) [file pone.0220695.s006.docx]

**S1 Table:** Indicator value, p value, frequency (number of times that the OTU was present among samples) and detailed taxonomy of fractions indicative OTUs.

| **NCBI Sequence** | **Fraction** | **Indicator value** | **P value** | **Frequency** | **Phyla** | **Class** | **Order** | **Family** | **Genus** | **Strain/Clone/Species** |
| --- | --- | --- | --- | --- | --- | --- | --- | --- | --- | --- |
| HQ114121 | FL | 0.92 | 0.001 | 9 | *Acidobacteria* | *Acidobacteria* | *Subgroup_6* | *uncultured_bacterium* | |  |
| KC554233 | FL | 0.90 | 0.001 | 14 | *Actinobacteria* | *Actinobacteria* | *Frankiales* | *Nakamurellaceae* | *Nakamurella* | *uncultured_bacterium* |
| KC554339 | FL | 0.88 | 0.002 | 19 | *Actinobacteria* | *MB-A2-108* | *uncultured_bacterium* | |  |  |
| JN656789 | FL | 0.87 | 0.002 | 21 | *Acidobacteria* | *Acidobacteria* | *Subgroup_6* | *uncultured_Acidobacteria_bacterium* | | |
| KC554912 | FL | 0.86 | 0.008 | 24 | *Actinobacteria* | *MB-A2-108* | *uncultured_bacterium* | |  |  |
| JF174954 | FL | 0.82 | 0.017 | 13 | *Actinobacteria* | *Actinobacteria* | *Corynebacteriales* | *Mycobacteriaceae* | *Mycobacterium* | *uncultured_bacterium* |
| JN869224 | FL | 0.80 | 0.049 | 20 | *Verrucomicrobia* | *Spartobacteria* | *Chthoniobacterales* | *Chthoniobacteraceae* | *Chthoniobacter* | *uncultured_bacterium* |
| JN869029 | FL | 0.79 | 0.001 | 16 | *Verrucomicrobia* | *Verrucomicrobiae* | *Verrucomicrobiales* | *Verrucomicrobiaceae* | *Prosthecobacter* | *uncultured_bacterium* |
| HM128683 | FL | 0.77 | 0.01 | 16 | *Actinobacteria* | *Acidimicrobiia* | *Acidimicrobiales* | *Acidimicrobiaceae* | *CL500-29_marine_group* | *uncultured_bacterium* |
| JN672135 | FL | 0.77 | 0.01 | 15 | *Proteobacteria* | *Alphaproteobacteria* | *Rhizobiales* | *Hyphomicrobiaceae* | *Hyphomicrobium* | *uncultured_bacterium* |
| EU803802 | FL | 0.77 | 0.003 | 3 | *Proteobacteria* | *Alphaproteobacteria* | *SAR11_clade* | *uncultured_bacterium* | |  |
| EU133219 | FL | 0.76 | 0.009 | 14 | *Actinobacteria* | *Thermoleophilia* | *Gaiellales* | *uncultured* | *uncultured_bacterium* | |
| EU592508 | FL | 0.76 | 0.028 | 17 | *Actinobacteria* | *Actinobacteria* | *Frankiales* | *Sporichthyaceae* | *hgcI_clade* | *uncultured_bacterium* |
| EU804053 | FL | 0.76 | 0.017 | 23 | *Proteobacteria* | *Alphaproteobacteria* | *SAR11_clade* | *LD12_freshwater_group* | *uncultured_bacterium* | |
| AM935724 | FL | 0.75 | 0.004 | 15 | *Chloroflexi* | *JG30-KF-CM66* | *uncultured_Chloroflexi_bacterium* | | | |
| HM275988 | FL | 0.75 | 0.001 | 8 | *Actinobacteria* | *Thermoleophilia* | *Solirubrobacterales* | *480-2* | *uncultured_bacterium* | |
| EU804028 | FL | 0.75 | 0.001 | 9 | *Actinobacteria* | *Acidimicrobiia* | *Acidimicrobiales* | *Acidimicrobiaceae* | *CL500-29_marine_group* | *uncultured_bacterium* |
| FN668062 | FL | 0.74 | 0.003 | 17 | *Proteobacteria* | *TA18* | *uncultured_delta_proteobacterium* | | | |
| AB672185 | FL | 0.71 | 0.023 | 4 | *Proteobacteria* | *Gammaproteobacteria* | *Oceanospirillales* | *Oceanospirillaceae* | *Pseudospirillum* | *uncultured_bacterium* |
| AF523901 | FL | 0.70 | 0.033 | 18 | *Cyanobacteria* | *ML635J-21* | *uncultured_bacterium* | |  |  |
| AM935269 | FL | 0.67 | 0.019 | 18 | *Chloroflexi* | *JG30-KF-CM66* | *uncultured_Chloroflexi_bacterium* | | | |
| GU305700 | FL | 0.67 | 0.001 | 6 | *Proteobacteria* | *Gammaproteobacteria* | *Legionellales* | *Legionellaceae* | *Legionella* | *uncultured_bacterium* |
| HQ114054 | FL | 0.66 | 0.016 | 20 | *Planctomycetes* | *OM190* | *uncultured_bacterium* | |  |  |
| JF800690 | FL | 0.66 | 0.005 | 23 | *SHA-109* | *uncultured_bacterium* | |  |  |  |
| JN941791 | FL | 0.65 | 0.013 | 5 | *Planctomycetes* | *Planctomycetacia* | *Planctomycetales* | *Planctomycetaceae* | *uncultured* | *uncultured_bacterium* |
| JN656931 | FL | 0.65 | 0.042 | 18 | *Chloroflexi* | *SL56_marine_group* | *uncultured_bacterium* | |  |  |
| EU133918 | FL | 0.61 | 0.017 | 10 | *Chlamydiae* | *Chlamydiae* | *Chlamydiales* | *Simkaniaceae* | *Candidatus_Rhabdochlamydia* | *uncultured_bacterium* |
| KC172329 | FL | 0.59 | 0.01 | 9 | *Proteobacteria* | *Alphaproteobacteria* | *Rhizobiales* | *Rhizobiales_Incertae_Sedis* | *Rhizomicrobium* | *uncultured_alpha_proteobacterium* |
| DQ520173 | FL | 0.59 | 0.014 | 12 | *Actinobacteria* | *Actinobacteria* | *Frankiales* | *Sporichthyaceae* | *hgcI_clade* | *uncultured_bacterium* |
| AJ565420 | FL | 0.58 | 0.007 | 5 | *Proteobacteria* | *Alphaproteobacteria* | *Sphingomonadales* | *7B-8* | *Sphingomonadaceae_bacterium_MWH-CaK2* | |
| JN391738 | FL | 0.57 | 0.016 | 14 | *Planctomycetes* | *Planctomycetacia* | *Planctomycetales* | *Planctomycetaceae* | *Planctomyces* | *uncultured_bacterium* |
| EF018643 | FL | 0.51 | 0.043 | 18 | *Acidobacteria* | *Acidobacteria* | *Subgroup_6* | *uncultured_bacterium* | |  |
| AB682299 | FL | 0.50 | 0.013 | 16 | *Proteobacteria* | *Betaproteobacteria* | *Burkholderiales* | *Burkholderiaceae* | *Limnobacter* | *Limnobacter_litoralis* |
| DQ676318 | FL | 0.50 | 0.014 | 2 | *Verrucomicrobia* | *OPB35_soil_group* | *uncultured_Verrucomicrobia_bacterium* | | | |
| GU940751 | FL | 0.50 | 0.019 | 13 | *Cyanobacteria* | *ML635J-21* | *uncultured_bacterium* | |  |  |
| AY922093 | FL | 0.49 | 0.023 | 13 | *Candidate_division_OD1* | *uncultured_Parcubacteria_bacterium* | | | |  |
| GQ007402 | FL | 0.48 | 0.044 | 5 | *Proteobacteria* | *Betaproteobacteria* | *Rhodocyclales* | *Rhodocyclaceae* | *12up* | *uncultured_bacterium* |
| GQ397007 | FL | 0.47 | 0.034 | 6 | *Chloroflexi* | *Thermomicrobia* | *JG30-KF-CM45* | *uncultured_bacterium* | |  |
| HQ860608 | FL | 0.42 | 0.034 | 4 | *Actinobacteria* | *Acidimicrobiia* | *Acidimicrobiales* | *Acidimicrobiaceae* | *CL500-29_marine_group* | *uncultured_bacterium* |
| JQ428024 | FL | 0.42 | 0.031 | 5 | *Proteobacteria* | *Alphaproteobacteria* | *Rhizobiales* | *Xanthobacteraceae* | *uncultured* | *uncultured_bacterium* |
| HQ597523 | FL | 0.42 | 0.039 | 6 | *Acidobacteria* | *Acidobacteria* | *Subgroup_6* | *uncultured_Acidobacteria_bacterium* | | |
| GU731298 | FL | 0.42 | 0.042 | 15 | *Proteobacteria* | *Alphaproteobacteria* | *Rhizobiales* | *Hyphomicrobiaceae* | *Devosia* | *bacterium_enrichment_culture_clone_heteroB88_4W* |
| GU563747 | FL | 0.42 | 0.031 | 11 | *Proteobacteria* | *Betaproteobacteria* | *Burkholderiales* | *Comamonadaceae* | *Curvibacter* | *uncultured_Curvibacter_sp.* |
| KF697487 | FL | 0.42 | 0.036 | 6 | *Acidobacteria* | *Acidobacteria* | *Subgroup_6* | *uncultured_bacterium* | |  |
| HM267237 | FL | 0.42 | 0.033 | 6 | *Candidate_division_TM7* | *uncultured_bacterium* | |  |  |  |
| JF904874 | FL | 0.42 | 0.037 | 3 | *Proteobacteria* | *Betaproteobacteria* | *Neisseriales* | *Neisseriaceae* | *Aquitalea* | *Aquitalea_sp._KJ011* |
| KC836077 | FL | 0.41 | 0.048 | 16 | *Proteobacteria* | *Betaproteobacteria* | *Burkholderiales* | *Alcaligenaceae* | *MWH-UniP1_aquatic_group* | *uncultured_bacterium* |
| KC836049 | PA | 0.91 | 0.001 | 17 | *Proteobacteria* | *Betaproteobacteria* | *Burkholderiales* | *Comamonadaceae* | *Sphaerotilus* | *uncultured_bacterium* |
| JN032909 | PA | 0.91 | 0.001 | 24 | *Planctomycetes* | *Phycisphaerae* | *Phycisphaerales* | *Phycisphaeraceae* | *CL500-3* | *uncultured_bacterium* |
| JN869043 | PA | 0.86 | 0.011 | 21 | *Planctomycetes* | *Planctomycetacia* | *Planctomycetales* | *Planctomycetaceae* | *uncultured* | *uncultured_bacterium* |
| FJ916091 | PA | 0.86 | 0.001 | 17 | *Bacteroidetes* | *Flavobacteriia* | *Flavobacteriales* | *NS9_marine_group* | *uncultured_Bacteroidetes_bacterium* | |
| EU801389 | PA | 0.84 | 0.003 | 22 | *Actinobacteria* | *Actinobacteria* | *Frankiales* | *Sporichthyaceae* | *hgcI_clade* | *uncultured_bacterium* |
| FJ830570 | PA | 0.83 | 0.003 | 23 | *Cyanobacteria* | *Cyanobacteria* | *SubsectionIV* | *FamilyI* | *Anabaena* | *Dolichospermum_circinale_CENA190* |
| KC253357 | PA | 0.83 | 0.002 | 17 | *Verrucomicrobia* | *Verrucomicrobia_Incertae_Sedis* | *Unknown_Order* | *Unknown_Family* | *Candidatus_Methylacidiphilum* | *uncultured_bacterium* |
| FJ612337 | PA | 0.81 | 0.009 | 24 | *Verrucomicrobia* | *Spartobacteria* | *Chthoniobacterales* | *FukuN18_freshwater_group* | *uncultured_bacterium* | |
| AF247591 | PA | 0.80 | 0.012 | 22 | *Cyanobacteria* | *Cyanobacteria* | *SubsectionIV* | *FamilyI* | *Anabaena* | *Anabaena_affinis_NIES-40* |
| GU305807 | PA | 0.79 | 0.004 | 11 | *Planctomycetes* | *Phycisphaerae* | *Phycisphaerales* | *Phycisphaeraceae* | *CL500-3* | *uncultured_bacterium* |
| HQ661200 | PA | 0.78 | 0.008 | 23 | *Actinobacteria* | *Actinobacteria* | *Frankiales* | *Sporichthyaceae* | *hgcI_clade* | *uncultured_bacterium* |
| EU803688 | PA | 0.78 | 0.001 | 23 | *Actinobacteria* | *Actinobacteria* | *Frankiales* | *Sporichthyaceae* | *hgcI_clade* | *uncultured_bacterium* |
| JF922442 | PA | 0.77 | 0.022 | 16 | *Chloroflexi* | *KD4-96* | *uncultured_bacterium* | |  |  |
| KC253307 | PA | 0.77 | 0.007 | 21 | *Proteobacteria* | *Betaproteobacteria* | *Burkholderiales* | *Burkholderiaceae* | *Limnobacter* | *uncultured_bacterium* |
| CU918341 | PA | 0.75 | 0.004 | 15 | *Actinobacteria* | *Actinobacteria* | *PeM15* | *uncultured_bacterium* | |  |
| JN868894 | PA | 0.75 | 0.003 | 18 | *Proteobacteria* | *Gammaproteobacteria* | *Alteromonadales* | *Alteromonadaceae* | *BD1-7_clade* | *uncultured_bacterium* |
| FJ830579 | PA | 0.74 | 0.005 | 22 | *Cyanobacteria* | *Cyanobacteria* | *SubsectionIV* | *FamilyI* | *Aphanizomenon* | *Anabaena_cf._fallax_CENA208* |
| EU803321 | PA | 0.73 | 0.011 | 19 | *Bacteroidetes* | *Cytophagia* | *Cytophagales* | *Cytophagaceae* | *uncultured* | *uncultured_bacterium* |
| EU803667 | PA | 0.72 | 0.019 | 10 | *Bacteroidetes* | *Sphingobacteriia* | *Sphingobacteriales* | *env.OPS_17* | *uncultured_bacterium* | |
| AY509523 | PA | 0.72 | 0.005 | 19 | *Acidobacteria* | *Acidobacteria* | *Subgroup_4* |  | *uncultured_Acidobacteria_bacterium* | |
| AB753965 | PA | 0.70 | 0.012 | 17 | *Verrucomicrobia* | *Spartobacteria* | *Chthoniobacterales* | *Chthoniobacteraceae* | *Chthoniobacter* | *uncultured_bacterium* |
| HM446115 | PA | 0.69 | 0.015 | 11 | *Actinobacteria* | *Actinobacteria* | *Frankiales* | *Sporichthyaceae* | *Sporichthya* | *uncultured_bacterium* |
| KC189665 | PA | 0.69 | 0.006 | 15 | *Proteobacteria* | *Deltaproteobacteria* | *Myxococcales* | *0319-6G20* | *uncultured_bacterium* | |
| GU305750 | PA | 0.69 | 0.029 | 20 | *Planctomycetes* | *Planctomycetacia* | *Planctomycetales* | *Planctomycetaceae* | *uncultured* | *uncultured_bacterium* |
| JN626564 | PA | 0.69 | 0.018 | 17 | *Actinobacteria* | *Actinobacteria* | *Micrococcales* | *Microbacteriaceae* | *Alpinimonas* | *uncultured_bacterium* |
| EU803309 | PA | 0.69 | 0.046 | 23 | *Actinobacteria* | *Acidimicrobiia* | *Acidimicrobiales* | *Acidimicrobiaceae* | *CL500-29_marine_group* | *uncultured_bacterium* |
| AY792234 | PA | 0.65 | 0.01 | 14 | *Actinobacteria* | *Acidimicrobiia* | *Acidimicrobiales* | *uncultured* | *uncultured_actinobacterium* | |
| AY947972 | PA | 0.65 | 0.011 | 4 | *Bacteroidetes* | *Flavobacteriia* | *Flavobacteriales* | *Cryomorphaceae* | *Fluviicola* | *uncultured_Bacteroidetes_bacterium* |
| JN868810 | PA | 0.65 | 0.01 | 5 | *Proteobacteria* | *Gammaproteobacteria* | *Pseudomonadales* | *Pseudomonadaceae* | *Pseudomonas* | *uncultured_bacterium* |
| JN606076 | PA | 0.65 | 0.001 | 5 | *Chlamydiae* | *Chlamydiae* | *Chlamydiales* | *Simkaniaceae* | *uncultured* | *Chlamydiales_bacterium_NS16* |
| HQ730087 | PA | 0.65 | 0.012 | 11 | *Cyanobacteria* | *Cyanobacteria* | *SubsectionIV* | *FamilyI* | *Sphaerospermopsis_torques-reginae_ITEP-026* | |
| EU803797 | PA | 0.65 | 0.049 | 24 | *Bacteroidetes* | *Sphingobacteriia* | *Sphingobacteriales* | *Chitinophagaceae* | *Ferruginibacter* | *uncultured_bacterium* |
| GU127190 | PA | 0.64 | 0.002 | 12 | *Proteobacteria* | *Gammaproteobacteria* | *Xanthomonadales* | *Xanthomonadales_Incertae_Sedis* | *uncultured* | *uncultured_bacterium* |
| KC682950 | PA | 0.64 | 0.029 | 15 | *Proteobacteria* | *Alphaproteobacteria* | *Rhizobiales* | *alphaI_cluster* | *uncultured_bacterium* | |
| HM270195 | PA | 0.64 | 0.016 | 5 | *Actinobacteria* | *Acidimicrobiia* | *Acidimicrobiales* | *uncultured* | *uncultured_bacterium* | |
| FJ612232 | PA | 0.63 | 0.016 | 2 | *Bacteroidetes* | *Flavobacteriia* | *Flavobacteriales* | *Cryomorphaceae* | *Fluviicola* | *uncultured_bacterium* |
| GU305698 | PA | 0.63 | 0.008 | 8 | *Verrucomicrobia* | *Spartobacteria* | *Chthoniobacterales* | *DA101_soil_group* | *uncultured_bacterium* | |
| JX505108 | PA | 0.62 | 0.026 | 20 | *Actinobacteria* | *Acidimicrobiia* | *Acidimicrobiales* | *uncultured* | *uncultured_Ferrimicrobium_sp.* | |
| GQ859644 | PA | 0.62 | 0.011 | 9 | *Cyanobacteria* | *Cyanobacteria* | *SubsectionIII* | *FamilyI* | *Pseudanabaena_mucicola_PMC279.06* | |
| JN941777 | PA | 0.62 | 0.018 | 13 | *Proteobacteria* | *Betaproteobacteria* | *Burkholderiales* | *uncultured* | *uncultured_bacterium* | |
| KC683058 | PA | 0.62 | 0.019 | 5 | *Proteobacteria* | *Alphaproteobacteria* | *Rhizobiales* | *JG35-K1-AG5* | *uncultured_bacterium* | |
| AB757748 | PA | 0.61 | 0.029 | 7 | *Planctomycetes* | *Planctomycetacia* | *Planctomycetales* | *Planctomycetaceae* | *uncultured* | *uncultured_bacterium* |
| JN626564 | PA | 0.59 | 0.05 | 20 | *Actinobacteria* | *Actinobacteria* | *Micrococcales* | *Microbacteriaceae* | *Alpinimonas* | *uncultured_bacterium* |
| HQ661200 | PA | 0.59 | 0.016 | 9 | *Actinobacteria* | *Actinobacteria* | *Frankiales* | *Sporichthyaceae* | *hgcI_clade* | *uncultured_bacterium* |
| EU134497 | PA | 0.58 | 0.006 | 5 | *Proteobacteria* | *Deltaproteobacteria* | *Bdellovibrionales* | *Bdellovibrionaceae* | *OM27_clade* | *uncultured_bacterium* |
| GU118331 | PA | 0.58 | 0.006 | 4 | *TM6* | *uncultured_bacterium* | |  |  |  |
| HQ827891 | PA | 0.58 | 0.009 | 13 | *Bacteroidetes* | *Cytophagia* | *Cytophagales* | *Cytophagaceae* | *uncultured* | *uncultured_bacterium* |
| GQ859616 | PA | 0.56 | 0.015 | 14 | *Cyanobacteria* | *Cyanobacteria* | *SubsectionIV* | *FamilyI* | *Anabaena_sphaerica_UTEX_'B_1616'* | |
| KC253272 | PA | 0.56 | 0.004 | 6 | *Proteobacteria* | *Deltaproteobacteria* | *Myxococcales* | *0319-6G20* | *uncultured_bacterium* | |
| KF287766 | PA | 0.54 | 0.042 | 8 | *Proteobacteria* | *Alphaproteobacteria* | *Rhodospirillales* | *I-10* | *uncultured_Oceanibaculum_sp.* | |
| EF540408 | PA | 0.51 | 0.022 | 7 | *Proteobacteria* | *Betaproteobacteria* | *Nitrosomonadales* | *Nitrosomonadaceae* | *uncultured* | *uncultured_soil_bacterium* |
| KC836016 | PA | 0.51 | 0.021 | 7 | *Proteobacteria* | *Betaproteobacteria* | *Burkholderiales* | *Comamonadaceae* | *uncultured* | *uncultured_bacterium* |
| HQ386626 | PA | 0.50 | 0.017 | 7 | *Cyanobacteria* | *Melainabacteria* | *Caenarcaniphilales* | *uncultured_cyanobacterium* | | |
| AF316773 | PA | 0.50 | 0.012 | 8 | *Planctomycetes* | *OM190* | *uncultured_Crater_Lake_bacterium_CL500-15* | | | |
| AF418968 | PA | 0.50 | 0.014 | 2 | *Planctomycetes* | *OM190* | *uncultured_bacterium* | |  |  |
| HM799060 | PA | 0.49 | 0.034 | 17 | *Cyanobacteria* | *Melainabacteria* | *Vampirovibrionales* | *uncultured_cyanobacterium* | | |
| DQ450182 | PA | 0.48 | 0.037 | 1 | *Bacteroidetes* | *Flavobacteriia* | *Flavobacteriales* | *Cryomorphaceae* | *Fluviicola* | *uncultured_proteobacterium* |
| AF207074 | PA | 0.47 | 0.039 | 2 | *Bacteroidetes* | *Flavobacteriia* | *Flavobacteriales* | *Flavobacteriaceae* | *Elizabethkingia* | *Elizabethkingia_meningoseptica* |
| DQ463716 | PA | 0.47 | 0.037 | 5 | *Bacteroidetes* | *Sphingobacteriia* | *Sphingobacteriales* | *ST-12K33* | *uncultured_Bacteroidetes_bacterium* | |
| JN868942 | PA | 0.46 | 0.03 | 3 | *Proteobacteria* | *Gammaproteobacteria* | *Legionellales* | *Legionellaceae* | *Legionella* | *uncultured_bacterium* |
| JQ814737 | PA | 0.45 | 0.047 | 5 | *Cyanobacteria* | *Cyanobacteria* | *SubsectionIII* | *FamilyI* | *Phormidium* | *uncultured_bacterium* |
| DQ856516 | PA | 0.45 | 0.044 | 6 | *Bacteroidetes* | *Sphingobacteriia* | *Sphingobacteriales* | *Chitinophagaceae* | *uncultured* | *uncultured_bacterium* |
| AB661545 | PA | 0.42 | 0.032 | 3 | *Proteobacteria* | *Alphaproteobacteria* | *Rhodospirillales* | *Rhodospirillaceae* | *Roseospirillum* | *uncultured_bacterium* |
| KF697437 | PA | 0.42 | 0.042 | 6 | *Proteobacteria* | *Gammaproteobacteria* | *Alteromonadales* | *Alteromonadaceae* | *BD1-7_clade* | *uncultured_bacterium* |
| GQ340071 | PA | 0.42 | 0.036 | 6 | *Bacteroidetes* | *Sphingobacteriia* | *Sphingobacteriales* | *env.OPS_17* | *uncultured_bacterium* | |
| EU135413 | PA | 0.42 | 0.032 | 3 | *Verrucomicrobia* | *Spartobacteria* | *Chthoniobacterales* | *Chthoniobacteraceae* | *Chthoniobacter* | *uncultured_bacterium* |
| FJ208823 | PA | 0.42 | 0.04 | 14 | *Planctomycetes* | *OM190* | *uncultured_bacterium* | |  |  |
| DQ520168 | PA | 0.42 | 0.032 | 2 | *Bacteroidetes* | *Sphingobacteriia* | *Sphingobacteriales* | *ST-12K33* | *uncultured_bacterium* | |
| AF236012 | PA | 0.42 | 0.046 | 6 | *Proteobacteria* | *Betaproteobacteria* | *Nitrosomonadales* | *Nitrosomonadaceae* | *uncultured* | *beta_proteobacterium_A0837* |
| AB753965 | PA | 0.42 | 0.044 | 4 | *Verrucomicrobia* | *Spartobacteria* | *Chthoniobacterales* | *Chthoniobacteraceae* | *Chthoniobacter* | *uncultured_bacterium* |
| KF460029 | PA | 0.42 | 0.042 | 6 | *Proteobacteria* | *Betaproteobacteria* | *Burkholderiales* | *Burkholderiaceae* | *Cupriavidus* | *Cupriavidus_sp._USMAA2-4* |
| AACY020170993 | PA | 0.42 | 0.029 | 2 | *Proteobacteria* | *Deltaproteobacteria* | *Myxococcales* | *0319-6G20* | *marine_metagenome* | |
| AY945863 | PA | 0.39 | 0.047 | 8 | *Proteobacteria* | *Betaproteobacteria* | *Rhodocyclales* | *Rhodocyclaceae* | *Thauera* | *uncultured_bacterium* |
