# Supplementary material for: Spatial heterogeneity and hydrological fluctuations drive bacterioplankton community composition in an Amazon floodplain system
Source: PLoS One. 2019 Aug 9;14(8):e0220695. doi: 10.1371/journal.pone.0220695 (PMC6688838; doi:10.1371/journal.pone.0220695)
Supplement: S2 Table — (DOCX) [file pone.0220695.s007.docx]

**S2 Table:** Indicator value, p value, frequency (number of times that the OTU was present among samples) and detailed taxonomy of hydrologic periods indicative OTUs.

| **NCBI Sequence** | **Period** | **Indicator value** | **P value** | **Frequency** | **Phyla** | **Class** | **Order** | **Family** | **Genus** | **Strain/Clone/Species** |
| --- | --- | --- | --- | --- | --- | --- | --- | --- | --- | --- |
| EU592508 | Rising | 0.87 | 0.002 | 17 | *Actinobacteria* | *Actinobacteria* | *Frankiales* | *Sporichthyaceae* | *hgcI_clade* | *uncultured_bacterium* |
| DQ520189 | Rising | 0.85 | 0.001 | 20 | *Spirochaetae* | *Spirochaetes* | *Spirochaetales* | *Leptospiraceae* | *Leptospira* | *uncultured_bacterium* |
| EU804053 | Rising | 0.85 | 0.001 | 23 | *Proteobacteria* | *Alphaproteobacteria* | *SAR11_clade* | *LD12_freshwater_group* | *uncultured_bacterium* | |
| D84522 | Rising | 0.84 | 0.003 | 8 | *Proteobacteria* | *Alphaproteobacteria* | *Sphingomonadales* | *Sphingomonadaceae* | *Sphingomonas* | *Sphingomonas_sp._MK347* |
| EU803894 | Rising | 0.84 | 0.001 | 24 | *Actinobacteria* | *Actinobacteria* | *Frankiales* | *Sporichthyaceae* | *hgcI_clade* | *uncultured_bacterium* |
| HM856377 | Rising | 0.84 | 0.003 | 22 | *Proteobacteria* | *Betaproteobacteria* | *Methylophilales* | *Methylophilaceae* | *LD28_freshwater_group* | *uncultured_Methylophilaceae_bacterium* |
| EU801940 | Rising | 0.84 | 0.001 | 23 | *Proteobacteria* | *Betaproteobacteria* | *Burkholderiales* | *Burkholderiaceae* | *Polynucleobacter* | *uncultured_bacterium* |
| EU801613 | Rising | 0.82 | 0.001 | 18 | *Proteobacteria* | *Betaproteobacteria* | *Burkholderiales* | *Oxalobacteraceae* | *Paucimonas* | *uncultured_bacterium* |
| HM322101 | Rising | 0.82 | 0.001 | 23 | *Actinobacteria* | *Actinobacteria* | *Frankiales* | *Sporichthyaceae* | *hgcI_clade* | *uncultured_bacterium* |
| JN656882 | Rising | 0.82 | 0.005 | 24 | *Bacteroidetes* | *Sphingobacteriia* | *Sphingobacteriales* | *Chitinophagaceae* | *Sediminibacterium* | *uncultured_Bacteroidetes_bacterium* |
| KC253296 | Rising | 0.80 | 0.001 | 24 | *Actinobacteria* | *Actinobacteria* | *Frankiales* | *Sporichthyaceae* | *hgcI_clade* | *uncultured_bacterium* |
| GU127184 | Rising | 0.79 | 0.003 | 16 | *Proteobacteria* | *Betaproteobacteria* | *Burkholderiales* | *Comamonadaceae* | *Variovorax* | *uncultured_bacterium* |
| GU127278 | Rising | 0.79 | 0.001 | 24 | *Actinobacteria* | *Actinobacteria* | *Frankiales* | *Sporichthyaceae* | *hgcI_clade* | *uncultured_bacterium* |
| EU803802 | Rising | 0.79 | 0.002 | 24 | *Proteobacteria* | *Alphaproteobacteria* | *SAR11_clade* | *uncultured_bacterium* | |  |
| EU803573 | Rising | 0.78 | 0.003 | 20 | *Proteobacteria* | *Betaproteobacteria* | *Burkholderiales* | *Comamonadaceae* | *Limnohabitans* | *uncultured_bacterium* |
| KC189777 | Rising | 0.78 | 0.018 | 15 | *Proteobacteria* | *Betaproteobacteria* | *TRA3-20* | *uncultured_bacterium* | |  |
| JN868868 | Rising | 0.77 | 0.027 | 14 | *Candidate_division_TM7* | *uncultured_bacterium* | |  |  |  |
| EU801319 | Rising | 0.77 | 0.002 | 20 | *Proteobacteria* | *Betaproteobacteria* | *Nitrosomonadales* | *Nitrosomonadaceae* | *uncultured* | *uncultured_bacterium* |
| AB672185 | Rising | 0.77 | 0.01 | 4 | *Proteobacteria* | *Gammaproteobacteria* | *Oceanospirillales* | *Oceanospirillaceae* | *Pseudospirillum* | *uncultured_bacterium* |
| EU803847 | Rising | 0.76 | 0.001 | 22 | *Actinobacteria* | *Actinobacteria* | *Frankiales* | *Sporichthyaceae* | *hgcI_clade* | *uncultured_bacterium* |
| AACY023437746 | Rising | 0.76 | 0.008 | 19 | *Proteobacteria* | *Betaproteobacteria* | *Burkholderiales* | *Comamonadaceae* | *uncultured* | *marine_metagenome* |
| JN869093 | Rising | 0.76 | 0.001 | 18 | *Proteobacteria* | *Gammaproteobacteria* | *Xanthomonadales* | *uncultured* | *uncultured_bacterium* | |
| KC253357 | Rising | 0.76 | 0.011 | 17 | *Verrucomicrobia* | *Verrucomicrobia_Incertae_Sedis* | *Unknown_Order* | *Unknown_Family* | *Candidatus_Methylacidiphilum* | *uncultured_bacterium* |
| KC253357 | Rising | 0.75 | 0.002 | 24 | *Verrucomicrobia* | *Verrucomicrobia_Incertae_Sedis* | *Unknown_Order* | *Unknown_Family* | *Candidatus_Methylacidiphilum* | *uncultured_bacterium* |
| KC253327 | Rising | 0.75 | 0.017 | 12 | *Proteobacteria* | *Gammaproteobacteria* | *Xanthomonadales* | *uncultured* | *uncultured_bacterium* | |
| EU803281 | Rising | 0.75 | 0.003 | 23 | *Actinobacteria* | *Actinobacteria* | *Frankiales* | *Sporichthyaceae* | *hgcI_clade* | *uncultured_bacterium* |
| EU803951 | Rising | 0.75 | 0.001 | 24 | *Proteobacteria* | *Betaproteobacteria* | *Burkholderiales* | *Comamonadaceae* | *uncultured* | *uncultured_bacterium* |
| EU804035 | Rising | 0.74 | 0.001 | 24 | *Chloroflexi* | *SL56_marine_group* | *uncultured_bacterium* | |  |  |
| JF429366 | Rising | 0.74 | 0.001 | 21 | *Proteobacteria* | *Betaproteobacteria* | *Burkholderiales* | *Oxalobacteraceae* | *Paucimonas* | *uncultured_bacterium* |
| JN626564 | Rising | 0.74 | 0.027 | 19 | *Actinobacteria* | *Actinobacteria* | *Micrococcales* | *Microbacteriaceae* | *Alpinimonas* | *uncultured_bacterium* |
| EU803917 | Rising | 0.74 | 0.001 | 24 | *Actinobacteria* | *Actinobacteria* | *Frankiales* | *Sporichthyaceae* | *hgcI_clade* | *uncultured_bacterium* |
| JN656931 | Rising | 0.73 | 0.001 | 18 | *Chloroflexi* | *SL56_marine_group* | *uncultured_bacterium* | |  |  |
| GU183621 | Rising | 0.72 | 0.005 | 16 | *Verrucomicrobia* | *OPB35_soil_group* | *uncultured_bacterium* | |  |  |
| EU803961 | Rising | 0.72 | 0.017 | 21 | *Actinobacteria* | *Actinobacteria* | *Micrococcales* | *Microbacteriaceae* | *Candidatus_Planktoluna* | *uncultured_bacterium* |
| EU803227 | Rising | 0.72 | 0.017 | 24 | *Actinobacteria* | *Actinobacteria* | *Frankiales* | *Sporichthyaceae* | *hgcI_clade* | *uncultured_bacterium* |
| KC253346 | Rising | 0.71 | 0.008 | 23 | *Actinobacteria* | *Acidimicrobiia* | *Acidimicrobiales* | *Acidimicrobiaceae* | *CL500-29_marine_group* | *uncultured_bacterium* |
| EU803741 | Rising | 0.71 | 0.001 | 23 | *Actinobacteria* | *Acidimicrobiia* | *Acidimicrobiales* | *Acidimicrobiaceae* | *CL500-29_marine_group* | *uncultured_bacterium* |
| KC835934 | Rising | 0.71 | 0.001 | 23 | *Actinobacteria* | *Actinobacteria* | *Frankiales* | *Sporichthyaceae* | *hgcI_clade* | *uncultured_bacterium* |
| EU803321 | Rising | 0.70 | 0.016 | 19 | *Bacteroidetes* | *Cytophagia* | *Cytophagales* | *Cytophagaceae* | *uncultured* | *uncultured_bacterium* |
| EU803898 | Rising | 0.70 | 0.004 | 10 | *Chloroflexi* | *SL56_marine_group* | *uncultured_bacterium* | |  |  |
| EU803415 | Rising | 0.70 | 0.002 | 20 | *Bacteroidetes* | *Sphingobacteriia* | *Sphingobacteriales* | *Chitinophagaceae* | *uncultured* | *uncultured_bacterium* |
| AY328773 | Rising | 0.69 | 0.014 | 14 | *Proteobacteria* | *SPOTSOCT00m83* | *uncultured_bacterium* | |  |  |
| JQ958638 | Rising | 0.69 | 0.005 | 17 | *Proteobacteria* | *Betaproteobacteria* | *Methylophilales* | *Methylophilaceae* | *uncultured* | *uncultured_bacterium* |
| EU800430 | Rising | 0.69 | 0.019 | 20 | *Acidobacteria* | *Acidobacteria* | *Subgroup_6* | *uncultured_bacterium* | |  |
| JF830210 | Rising | 0.69 | 0.042 | 22 | *Proteobacteria* | *Betaproteobacteria* | *Burkholderiales* | *Alcaligenaceae* | *MWH-UniP1_aquatic_group* | *bacterium_enrichment_culture_clone_B63(2011)* |
| EU803802 | Rising | 0.69 | 0.017 | 3 | *Proteobacteria* | *Alphaproteobacteria* | *SAR11_clade* | *uncultured_bacterium* | |  |
| JN398062 | Rising | 0.68 | 0.021 | 16 | *Proteobacteria* | *Betaproteobacteria* | *Burkholderiales* | *Comamonadaceae* | *uncultured* | *uncultured_bacterium* |
| FJ184387 | Rising | 0.67 | 0.002 | 10 | *Cyanobacteria* | *Cyanobacteria* | *SubsectionIII* | *FamilyI* | *Planktothrix* | *Planktothrix_pseudagardhii_HAB366* |
| EU090709 | Rising | 0.67 | 0.002 | 6 | *Chlamydiae* | *Chlamydiae* | *Chlamydiales* | *Simkaniaceae* | *Candidatus_Rhabdochlamydia* | *uncultured_Candidatus_Rhabdochlamydia_sp.* |
| AM935269 | Rising | 0.65 | 0.038 | 18 | *Chloroflexi* | *JG30-KF-CM66* | *uncultured_Chloroflexi_bacterium* | | | |
| EU803777 | Rising | 0.64 | 0.021 | 14 | *Gemmatimonadetes* | *Gemmatimonadetes* | *Gemmatimonadales* | *Gemmatimonadaceae* | *uncultured* | *uncultured_bacterium* |
| KC253289 | Rising | 0.62 | 0.027 | 10 | *Proteobacteria* | *Deltaproteobacteria* | *Myxococcales* | *0319-6G20* | *uncultured_bacterium* | |
| DQ395963 | Rising | 0.62 | 0.026 | 16 | *Acidobacteria* | *Acidobacteria* | *Subgroup_6* | *uncultured_organism* | |  |
| EU683887 | Rising | 0.59 | 0.019 | 9 | *Chlamydiae* | *Chlamydiae* | *Chlamydiales* | *Simkaniaceae* | *Candidatus_Rhabdochlamydia* | *Chlamydiales_bacterium_CRIB33* |
| FJ894109 | Rising | 0.58 | 0.004 | 5 | *Proteobacteria* | *Gammaproteobacteria* | *Pseudomonadales* | *Pseudomonadaceae* | *Pseudomonas* | *uncultured_bacterium* |
| AY947972 | Rising | 0.58 | 0.041 | 4 | *Bacteroidetes* | *Flavobacteriia* | *Flavobacteriales* | *Cryomorphaceae* | *Fluviicola* | *uncultured_Bacteroidetes_bacterium* |
| EU133918 | Rising | 0.55 | 0.037 | 10 | *Chlamydiae* | *Chlamydiae* | *Chlamydiales* | *Simkaniaceae* | *Candidatus_Rhabdochlamydia* | *uncultured_bacterium* |
| AM935541 | Rising | 0.54 | 0.014 | 7 | *Proteobacteria* | *Betaproteobacteria* | *Nitrosomonadales* | *Nitrosomonadaceae* | *uncultured* | *uncultured_beta_proteobacterium* |
| EU133526 | Rising | 0.54 | 0.05 | 9 | *Proteobacteria* | *Alphaproteobacteria* | *Rhodospirillales* | *Rhodospirillaceae* | *uncultured* | *uncultured_bacterium* |
| JN606076 | Rising | 0.53 | 0.016 | 5 | *Chlamydiae* | *Chlamydiae* | *Chlamydiales* | *Simkaniaceae* | *uncultured* | *Chlamydiales_bacterium_NS16* |
| KC172329 | Rising | 0.53 | 0.027 | 9 | *Proteobacteria* | *Alphaproteobacteria* | *Rhizobiales* | *Rhizobiales_Incertae_Sedis* | *Rhizomicrobium* | *uncultured_alpha_proteobacterium* |
| DQ316339 | Rising | 0.53 | 0.042 | 8 | *Actinobacteria* | *Actinobacteria* | *Frankiales* | *Sporichthyaceae* | *hgcI_clade* | *uncultured_actinobacterium* |
| EU683887 | Rising | 0.51 | 0.034 | 8 | *Chlamydiae* | *Chlamydiae* | *Chlamydiales* | *Simkaniaceae* | *Candidatus_Rhabdochlamydia* | *Chlamydiales_bacterium_CRIB33* |
| AB682299 | Rising | 0.50 | 0.012 | 16 | *Proteobacteria* | *Betaproteobacteria* | *Burkholderiales* | *Burkholderiaceae* | *Limnobacter* | *Limnobacter_litoralis* |
| EF173340 | Rising | 0.50 | 0.01 | 9 | *Proteobacteria* | *Alphaproteobacteria* | *Rhodospirillales* | *Rhodospirillaceae* | *uncultured* | *uncultured_bacterium* |
| EU090707 | Rising | 0.47 | 0.014 | 7 | *Chlamydiae* | *Chlamydiae* | *Chlamydiales* | *Simkaniaceae* | *Candidatus_Rhabdochlamydia* | *uncultured_Candidatus_Rhabdochlamydia_sp.* |
| FR714402 | Rising | 0.46 | 0.037 | 3 | *Chlamydiae* | *Chlamydiae* | *Chlamydiales* | *Simkaniaceae* | *uncultured* | *uncultured_Chlamydia_sp.* |
| FJ936764 | Rising | 0.45 | 0.036 | 8 | *Proteobacteria* | *Alphaproteobacteria* | *Rhodospirillales* | *DA111* | *uncultured_bacterium* | |
| GQ340273 | Rising | 0.44 | 0.034 | 6 | *Proteobacteria* | *Alphaproteobacteria* | *Rhodospirillales* | *Rhodospirillaceae* | *Ferrovibrio* | *uncultured_bacterium* |
| DQ903988 | Rising | 0.42 | 0.036 | 7 | *Chlamydiae* | *Chlamydiae* | *Chlamydiales* | *Simkaniaceae* | *Candidatus_Rhabdochlamydia* | *uncultured_Chlamydia_sp.* |
| DQ903988 | Rising | 0.42 | 0.039 | 4 | *Chlamydiae* | *Chlamydiae* | *Chlamydiales* | *Simkaniaceae* | *Candidatus_Rhabdochlamydia* | *uncultured_Chlamydia_sp.* |
| AM991246 | Rising | 0.42 | 0.041 | 4 | *Proteobacteria* | *Alphaproteobacteria* | *Rickettsiales* | *mitochondria* | *uncultured_bacterium* | |
| GU305732 | Falling | 0.96 | 0.001 | 22 | *Planctomycetes* | *Planctomycetacia* | *Planctomycetales* | *Planctomycetaceae* | *Pirellula* | *uncultured_bacterium* |
| GQ340346 | Falling | 0.94 | 0.001 | 22 | *Planctomycetes* | *Planctomycetacia* | *Planctomycetales* | *Planctomycetaceae* | *Planctomyces* | *uncultured_bacterium* |
| BX294853 | Falling | 0.94 | 0.001 | 20 | *Planctomycetes* | *Planctomycetacia* | *Planctomycetales* | *Planctomycetaceae* | *Planctomyces* | *uncultured_bacterium* |
| AF239694 | Falling | 0.94 | 0.002 | 18 | *Planctomycetes* | *Planctomycetacia* | *Planctomycetales* | *Planctomycetaceae* | *Gemmata* | *Gemmata-like_str._JW3-8s0* |
| HQ661209 | Falling | 0.94 | 0.001 | 22 | *Cyanobacteria* | *Cyanobacteria* | *SubsectionI* | *FamilyI* | *Synechococcus* | *uncultured_bacterium* |
| DQ444388 | Falling | 0.93 | 0.001 | 24 | *Planctomycetes* | *Planctomycetacia* | *Planctomycetales* | *Planctomycetaceae* | *Planctomyces* | *uncultured_bacterium* |
| KC835930 | Falling | 0.93 | 0.005 | 24 | *Cyanobacteria* | *Cyanobacteria* | *SubsectionI* | *FamilyI* | *Microcystis* | *uncultured_bacterium* |
| AB661594 | Falling | 0.93 | 0.001 | 23 | *Cyanobacteria* | *Cyanobacteria* | *SubsectionI* | *FamilyI* | *uncultured_bacterium* | |
| AM710363 | Falling | 0.92 | 0.001 | 24 | *Cyanobacteria* | *Cyanobacteria* | *SubsectionI* | *FamilyI* | *Merismopedia* | *Cyanobium_sp._JJ2-3* |
| EU340186 | Falling | 0.92 | 0.002 | 24 | *Actinobacteria* | *Acidimicrobiia* | *Acidimicrobiales* | *uncultured* | *uncultured_bacterium* | |
| GU305789 | Falling | 0.92 | 0.001 | 19 | *Planctomycetes* | *Planctomycetacia* | *Planctomycetales* | *Planctomycetaceae* | *Planctomyces* | *uncultured_bacterium* |
| JN679170 | Falling | 0.91 | 0.001 | 20 | *Armatimonadetes* | *Armatimonadia* | *Armatimonadales* | *Armatimonadaceae* | *Armatimonas* | *uncultured_beta_proteobacterium* |
| HM127635 | Falling | 0.90 | 0.001 | 24 | *Cyanobacteria* | *Cyanobacteria* | *SubsectionI* | *FamilyI* | *Synechococcus* | *uncultured_bacterium* |
| KC620924 | Falling | 0.90 | 0.001 | 10 | *Proteobacteria* | *Alphaproteobacteria* | *Rhizobiales* | *Hyphomicrobiaceae* | *Hyphomicrobium* | *uncultured_bacterium* |
| AF316773 | Falling | 0.88 | 0.002 | 23 | *Planctomycetes* | *OM190* | *uncultured_Crater_Lake_bacterium_CL500-15* | | | |
| JX271902 | Falling | 0.87 | 0.01 | 16 | *Actinobacteria* | *Acidimicrobiia* | *Acidimicrobiales* | *Acidimicrobiaceae* | *CL500-29_marine_group* | *uncultured_bacterium* |
| JN656817 | Falling | 0.87 | 0.001 | 21 | *Bacteroidetes* | *Sphingobacteriia* | *Sphingobacteriales* | *Chitinophagaceae* | *Ferruginibacter* | *uncultured_Bacteroidetes_bacterium* |
| JF830148 | Falling | 0.86 | 0.017 | 23 | *Planctomycetes* | *Planctomycetacia* | *Planctomycetales* | *Planctomycetaceae* | *uncultured* | *uncultured_bacterium* |
| EU644173 | Falling | 0.86 | 0.005 | 14 | *Actinobacteria* | *Acidimicrobiia* | *Acidimicrobiales* | *uncultured* | *uncultured_bacterium* | |
| HE648177 | Falling | 0.85 | 0.009 | 22 | *Verrucomicrobia* | *OPB35_soil_group* | *uncultured_Verrucomicrobia_subdivision_3_bacterium* | | | |
| JF830208 | Falling | 0.85 | 0.001 | 19 | *Planctomycetes* | *Planctomycetacia* | *Planctomycetales* | *Planctomycetaceae* | *Blastopirellula* | *bacterium_enrichment_culture_clone_B55(2011)* |
| KC253366 | Falling | 0.85 | 0.004 | 24 | *Cyanobacteria* | *Cyanobacteria* | *SubsectionI* | *FamilyI* | *Synechococcus* | *uncultured_bacterium* |
| AY212628 | Falling | 0.84 | 0.006 | 21 | *Armatimonadetes* | *Armatimonadia* | *Armatimonadales* | *Armatimonadaceae* | *Armatimonas* | *uncultured_bacterium* |
| JN868815 | Falling | 0.84 | 0.013 | 13 | *Planctomycetes* | *Planctomycetacia* | *Planctomycetales* | *Planctomycetaceae* | *uncultured* | *uncultured_bacterium* |
| DQ444452 | Falling | 0.84 | 0.001 | 23 | *Planctomycetes* | *Planctomycetacia* | *Planctomycetales* | *Planctomycetaceae* | *uncultured* | *uncultured_bacterium* |
| KC253336 | Falling | 0.83 | 0.005 | 24 | *Bacteroidetes* | *Cytophagia* | *Cytophagales* | *Cytophagaceae* | *Hymenobacter* | *uncultured_bacterium* |
| FJ382111 | Falling | 0.82 | 0.001 | 8 | *Cyanobacteria* | *Cyanobacteria* | *SubsectionI* | *FamilyI* | *Synechococcus* | *uncultured_bacterium* |
| JN869039 | Falling | 0.82 | 0.02 | 24 | *Planctomycetes* | *Planctomycetacia* | *Planctomycetales* | *Planctomycetaceae* | *uncultured* | *uncultured_bacterium* |
| GU305751 | Falling | 0.82 | 0.002 | 21 | *Planctomycetes* | *Planctomycetacia* | *Planctomycetales* | *Planctomycetaceae* | *Blastopirellula* | *uncultured_bacterium* |
| CU926364 | Falling | 0.81 | 0.001 | 8 | *Proteobacteria* | *Alphaproteobacteria* | *Rhizobiales* | *Hyphomicrobiaceae* | *Pedomicrobium* | *uncultured_bacterium* |
| JF922442 | Falling | 0.81 | 0.003 | 16 | *Chloroflexi* | *KD4-96* | *uncultured_bacterium* | |  |  |
| GU305750 | Falling | 0.81 | 0.003 | 20 | *Planctomycetes* | *Planctomycetacia* | *Planctomycetales* | *Planctomycetaceae* | *uncultured* | *uncultured_bacterium* |
| HM069053 | Falling | 0.80 | 0.004 | 19 | *Proteobacteria* | *Alphaproteobacteria* | *Rhizobiales* | *MNG7* | *uncultured_bacterium* | |
| KC157044 | Falling | 0.80 | 0.006 | 20 | *Bacteroidetes* | *Sphingobacteriia* | *Sphingobacteriales* | *Chitinophagaceae* | *uncultured* | *Niastella_sp._HME8655* |
| JF830203 | Falling | 0.79 | 0.001 | 19 | *Proteobacteria* | *Deltaproteobacteria* | *GR-WP33-30* | *bacterium_enrichment_culture_clone_B30(2011)* | | |
| JN038791 | Falling | 0.79 | 0.013 | 17 | *Candidate_division_WS3* | *uncultured_Latescibacteria_bacterium* | | | |  |
| JX505108 | Falling | 0.78 | 0.005 | 20 | *Actinobacteria* | *Acidimicrobiia* | *Acidimicrobiales* | *uncultured* | *uncultured_Ferrimicrobium_sp.* | |
| AF418950 | Falling | 0.78 | 0.024 | 20 | *Planctomycetes* | *Phycisphaerae* | *Phycisphaerales* | *Phycisphaeraceae* | *CL500-3* | *uncultured_bacterium* |
| KC331285 | Falling | 0.78 | 0.013 | 24 | *Chloroflexi* | *KD4-96* | *uncultured_bacterium* | |  |  |
| HM263209 | Falling | 0.77 | 0.004 | 16 | *Actinobacteria* | *Acidimicrobiia* | *Acidimicrobiales* | *Acidimicrobiales_Incertae_Sedis* | *Candidatus_Microthrix* | *uncultured_bacterium* |
| AB661543 | Falling | 0.77 | 0.001 | 9 | *Bacteroidetes* | *Sphingobacteriia* | *Sphingobacteriales* | *AKYH767* | *uncultured_bacterium* | |
| AF247591 | Falling | 0.76 | 0.015 | 22 | *Cyanobacteria* | *Cyanobacteria* | *SubsectionIV* | *FamilyI* | *Anabaena* | *Anabaena_affinis_NIES-40* |
| KF836214 | Falling | 0.75 | 0.001 | 3 | *Proteobacteria* | *Alphaproteobacteria* | *Caulobacterales* | *Hyphomonadaceae* | *uncultured* | *uncultured_bacterium* |
| HQ114058 | Falling | 0.73 | 0.015 | 20 | *Planctomycetes* | *Planctomycetacia* | *Planctomycetales* | *Planctomycetaceae* | *uncultured* | *uncultured_bacterium* |
| EU135040 | Falling | 0.72 | 0.006 | 7 | *Planctomycetes* | *Phycisphaerae* | *Phycisphaerales* | *Phycisphaeraceae* | *SM1A02* | *uncultured_bacterium* |
| JN868894 | Falling | 0.70 | 0.008 | 18 | *Proteobacteria* | *Gammaproteobacteria* | *Alteromonadales* | *Alteromonadaceae* | *BD1-7_clade* | *uncultured_bacterium* |
| GU454869 | Falling | 0.69 | 0.02 | 17 | *Acidobacteria* | *Acidobacteria* | *Subgroup_3* | *SJA-149* | *uncultured_bacterium* | |
| DQ520172 | Falling | 0.69 | 0.017 | 10 | *Actinobacteria* | *Acidimicrobiia* | *Acidimicrobiales* | *uncultured* | *uncultured_bacterium* | |
| JF830212 | Falling | 0.69 | 0.008 | 6 | *Planctomycetes* | *Planctomycetacia* | *Planctomycetales* | *Planctomycetaceae* | *Pirellula* | *bacterium_enrichment_culture_clone_B70(2011)* |
| JN868746 | Falling | 0.69 | 0.026 | 23 | *Acidobacteria* | *Acidobacteria* | *Subgroup_4* | *Unknown_Family* | *Blastocatella* | *uncultured_bacterium* |
| HM445220 | Falling | 0.69 | 0.018 | 23 | *Bacteroidetes* | *Sphingobacteriia* | *Sphingobacteriales* | *AKYH767* | *uncultured_bacterium* | |
| JF922553 | Falling | 0.68 | 0.017 | 13 | *Proteobacteria* | *Betaproteobacteria* | *Burkholderiales* | *Burkholderiaceae* | *Lautropia* | *uncultured_bacterium* |
| GQ397007 | Falling | 0.67 | 0.01 | 3 | *Chloroflexi* | *Thermomicrobia* | *JG30-KF-CM45* | *uncultured_bacterium* | |  |
| HM446115 | Falling | 0.67 | 0.041 | 11 | *Actinobacteria* | *Actinobacteria* | *Frankiales* | *Sporichthyaceae* | *Sporichthya* | *uncultured_bacterium* |
| FJ830579 | Falling | 0.66 | 0.027 | 22 | *Cyanobacteria* | *Cyanobacteria* | *SubsectionIV* | *FamilyI* | *Aphanizomenon* | *Anabaena_cf._fallax_CENA208* |
| AB757748 | Falling | 0.65 | 0.006 | 7 | *Planctomycetes* | *Planctomycetacia* | *Planctomycetales* | *Planctomycetaceae* | *uncultured* | *uncultured_bacterium* |
| AM935786 | Falling | 0.64 | 0.039 | 13 | *Actinobacteria* | *Acidimicrobiia* | *Acidimicrobiales* | *uncultured* | *uncultured_Acidimicrobidae_bacterium* | |
| AY509523 | Falling | 0.64 | 0.03 | 19 | *Acidobacteria* | *Acidobacteria* | *Subgroup_4* |  | *uncultured_Acidobacteria_bacterium* | |
| HQ114142 | Falling | 0.64 | 0.023 | 21 | *Verrucomicrobia* | *OPB35_soil_group* | *uncultured_bacterium* | |  |  |
| EU283346 | Falling | 0.64 | 0.005 | 5 | *Proteobacteria* | *Alphaproteobacteria* | *Rhizobiales* | *A0839* | *uncultured_gamma_proteobacterium* | |
| JN038717 | Falling | 0.63 | 0.023 | 15 | *Candidate_division_WS3* | *uncultured_Latescibacteria_bacterium* | | | |  |
| JQ941791 | Falling | 0.62 | 0.028 | 14 | *Verrucomicrobia* | *OPB35_soil_group* | *uncultured_bacterium* | |  |  |
| CU918341 | Falling | 0.61 | 0.047 | 15 | *Actinobacteria* | *Actinobacteria* | *PeM15* | *uncultured_bacterium* | |  |
| KC253276 | Falling | 0.61 | 0.04 | 6 | *Bacteroidetes* | *Sphingobacteriia* | *Sphingobacteriales* | *Chitinophagaceae* | *uncultured* | *uncultured_bacterium* |
| GQ859644 | Falling | 0.60 | 0.02 | 9 | *Cyanobacteria* | *Cyanobacteria* | *SubsectionIII* | *FamilyI* | *Pseudanabaena_mucicola_PMC279.06* | |
| JF830229 | Falling | 0.59 | 0.031 | 16 | *Planctomycetes* | *Planctomycetacia* | *Planctomycetales* | *Planctomycetaceae* | *Pirellula* | *bacterium_enrichment_culture_clone_B176(2011)* |
| AY947978 | Falling | 0.59 | 0.034 | 6 | *Bacteroidetes* | *Flavobacteriia* | *Flavobacteriales* | *NS9_marine_group* | *uncultured_Bacteroidetes_bacterium* | |
| DQ640688 | Falling | 0.58 | 0.006 | 7 | *Bacteroidetes* | *Sphingobacteriia* | *Sphingobacteriales* | *Saprospiraceae* | *uncultured* | *uncultured_Bacteroidetes_bacterium* |
| KF411639 | Falling | 0.58 | 0.007 | 5 | *Actinobacteria* | *Acidimicrobiia* | *Acidimicrobiales* | *Acidimicrobiales_Incertae_Sedis* | *Candidatus_Microthrix* | *uncultured_actinobacterium* |
| KC172272 | Falling | 0.58 | 0.01 | 13 | *Proteobacteria* | *Deltaproteobacteria* | *Myxococcales* | *Polyangiaceae* | *Sorangium* | *uncultured_delta_proteobacterium* |
| JN023788 | Falling | 0.58 | 0.031 | 12 | *Proteobacteria* | *Alphaproteobacteria* | *Rhizobiales* | *Hyphomicrobiaceae* | *Hyphomicrobium* | *uncultured_bacterium* |
| FM201077 | Falling | 0.57 | 0.015 | 17 | *Verrucomicrobia* | *OPB35_soil_group* | *uncultured_bacterium* | |  |  |
| JN868850 | Falling | 0.57 | 0.018 | 17 | *Planctomycetes* | *Phycisphaerae* | *Phycisphaerales* | *Phycisphaeraceae* | *CL500-3* | *uncultured_bacterium* |
| FJ769516 | Falling | 0.57 | 0.022 | 5 | *Planctomycetes* | *Planctomycetacia* | *Planctomycetales* | *Planctomycetaceae* | *Planctomyces* | *uncultured_bacterium* |
| FJ936832 | Falling | 0.57 | 0.007 | 19 | *Planctomycetes* | *Phycisphaerae* | *Phycisphaerales* | *Phycisphaeraceae* | *SM1A02* | *uncultured_bacterium* |
| KC189789 | Falling | 0.56 | 0.029 | 20 | *Actinobacteria* | *Acidimicrobiia* | *Acidimicrobiales* | *Acidimicrobiaceae* | *CL500-29_marine_group* | *uncultured_bacterium* |
| JQ794638 | Falling | 0.56 | 0.008 | 4 | *Proteobacteria* | *Deltaproteobacteria* | *Myxococcales* | *mle1-27* | *uncultured_Myxococcales_bacterium* | |
| HQ682028 | Falling | 0.55 | 0.006 | 7 | *Proteobacteria* | *Betaproteobacteria* | *Burkholderiales* | *Comamonadaceae* | *uncultured* | *uncultured_bacterium* |
| HQ661210 | Falling | 0.55 | 0.04 | 6 | *Armatimonadetes* | *uncultured_bacterium* | |  |  |  |
| JN391737 | Falling | 0.53 | 0.019 | 13 | *Verrucomicrobia* | *Verrucomicrobiae* | *Verrucomicrobiales* | *Verrucomicrobiaceae* | *Prosthecobacter* | *uncultured_bacterium* |
| AY375088 | Falling | 0.52 | 0.04 | 5 | *Bacteroidetes* | *Sphingobacteriia* | *Sphingobacteriales* | *Chitinophagaceae* | *Lacibacter* | *uncultured_bacterium* |
| FJ208845 | Falling | 0.51 | 0.049 | 14 | *Planctomycetes* | *OM190* | *uncultured_bacterium* | |  |  |
| AB274849 | Falling | 0.51 | 0.036 | 10 | *Proteobacteria* | *Alphaproteobacteria* | *Rhodospirillales* | *Acetobacteraceae* | *uncultured* | *uncultured_bacterium* |
| FJ828506 | Falling | 0.50 | 0.021 | 7 | *Planctomycetes* | *Planctomycetacia* | *Planctomycetales* | *Planctomycetaceae* | *uncultured* | *bacterium_enrichment_culture_clone_KIST-JJY167* |
| AF235999 | Falling | 0.50 | 0.014 | 5 | *Proteobacteria* | *Alphaproteobacteria* | *Rhizobiales* | *Xanthobacteraceae* | *Labrys* | *alpha_proteobacterium_A0838* |
| GU127204 | Falling | 0.50 | 0.016 | 8 | *Proteobacteria* | *Gammaproteobacteria* | *Methylococcales* | *Methylococcaceae* | *Methylocaldum* | *uncultured_Methylocaldum_sp.* |
| FJ612230 | Falling | 0.50 | 0.014 | 7 | *Cyanobacteria* | *Cyanobacteria* | *SubsectionI* | *FamilyI* | *Snowella* | *uncultured_bacterium* |
| EF516185 | Falling | 0.50 | 0.013 | 7 | *Proteobacteria* | *Deltaproteobacteria* | *Myxococcales* | *mle1-27* | *uncultured_bacterium* | |
| GQ859616 | Falling | 0.48 | 0.036 | 14 | *Cyanobacteria* | *Cyanobacteria* | *SubsectionIV* | *FamilyI* | *Anabaena_sphaerica_UTEX_'B_1616'* | |
| GQ340157 | Falling | 0.48 | 0.047 | 7 | *Proteobacteria* | *Betaproteobacteria* | *Burkholderiales* | *Burkholderiaceae* | *Lautropia* | *uncultured_bacterium* |
| HM329700 | Falling | 0.48 | 0.036 | 2 | *Chloroflexi* | *TK10* | *uncultured_bacterium* | |  |  |
| JQ923788 | Falling | 0.48 | 0.049 | 6 | *Proteobacteria* | *Alphaproteobacteria* | *Rhizobiales* | *Methylobacteriaceae* | *Meganema* | *uncultured_bacterium* |
| AF448072 | Falling | 0.47 | 0.044 | 6 | *Cyanobacteria* | *Cyanobacteria* | *SubsectionI* | *FamilyI* | *Cyanobium_sp._PCC_8966* | |
| FJ444644 | Falling | 0.42 | 0.034 | 5 | *Proteobacteria* | *Deltaproteobacteria* | *Myxococcales* | *Haliangiaceae* | *Haliangium* | *uncultured_bacterium* |
| EU135194 | Falling | 0.42 | 0.042 | 2 | *Planctomycetes* | *Planctomycetacia* | *Planctomycetales* | *Planctomycetaceae* | *Planctomyces* | *uncultured_bacterium* |
| KC874430 | Falling | 0.42 | 0.037 | 4 | *Planctomycetes* | *Phycisphaerae* | *Phycisphaerales* | *Phycisphaeraceae* | *SM1A02* | *uncultured_bacterium* |
| KC011149 | Falling | 0.42 | 0.037 | 4 | *Proteobacteria* | *Deltaproteobacteria* | *Myxococcales* | *Polyangiaceae* | *uncultured_delta_proteobacterium* | |
| JN868880 | Falling | 0.42 | 0.039 | 7 | *Acidobacteria* | *Subgroup_22* | *uncultured_bacterium* | |  |  |
| FJ502252 | Falling | 0.42 | 0.034 | 6 | *Bacteroidetes* | *Sphingobacteriia* | *Sphingobacteriales* | *Chitinophagaceae* | *Filimonas* | *uncultured_bacterium* |
| GQ480086 | Falling | 0.42 | 0.035 | 2 | *Planctomycetes* | *OM190* | *uncultured_bacterium* | |  |  |
| JX079242 | Falling | 0.42 | 0.031 | 5 | *Proteobacteria* | *Deltaproteobacteria* | *Myxococcales* | *Phaselicystidaceae* | *Phaselicystis* | *uncultured_bacterium* |
| DQ444389 | Falling | 0.40 | 0.044 | 4 | *Planctomycetes* | *Planctomycetacia* | *Planctomycetales* | *Planctomycetaceae* | *Planctomyces* | *uncultured_bacterium* |
| AF418968 | Falling | 0.39 | 0.038 | 2 | *Planctomycetes* | *OM190* | *uncultured_bacterium* | |  |  |
