# Supplementary material for: Spatial heterogeneity and hydrological fluctuations drive bacterioplankton community composition in an Amazon floodplain system
Source: PLoS One. 2019 Aug 9;14(8):e0220695. doi: 10.1371/journal.pone.0220695 (PMC6688838; doi:10.1371/journal.pone.0220695)
Supplement: S3 Table — (DOCX) [file pone.0220695.s008.docx]

**S3 Table:** Indicator value, p value, frequency (number of times that the OTU was present among samples) and detailed taxonomy of influence area indicative OTUs.

| **NCBI Sequence** | **Influence area** | **Indicator value** | **P value** | **Frequency** | **Phyla** | **Class** | **Order** | **Family** | **Genus** | **Strain/Clone/Species** | |
| --- | --- | --- | --- | --- | --- | --- | --- | --- | --- | --- | --- |
| GQ350701 | ATTZ | 0.62 | 0.043 | 11 | *Cyanobacteria* | *ML635J-21* | *uncultured_bacterium* | |  |  |  |
| JN941791 | ATTZ | 0.61 | 0.033 | 5 | *Planctomycetes* | *Planctomycetacia* | *Planctomycetales* | *Planctomycetaceae* | *uncultured* | *uncultured_bacterium* | |
| GQ472429 | ATTZ | 0.58 | 0.028 | 7 | *Proteobacteria* | *Alphaproteobacteria* | *Rhodospirillales* | *Acetobacteraceae* | *Acidocella* | *uncultured_bacterium* | |
| AY948005 | ATTZ | 0.58 | 0.05 | 22 | *Actinobacteria* | *Actinobacteria* | *Frankiales* | *Sporichthyaceae* | *hgcI_clade* | *uncultured_actinobacterium* | |
| KC432462 | Lake | 0.68 | 0.036 | 12 | *Proteobacteria* | *Betaproteobacteria* | *Burkholderiales* | *Comamonadaceae* | *Delftia* | *uncultured_bacterium* | |
| JN618338 | Lake | 0.57 | 0.031 | 11 | *Proteobacteria* | *Alphaproteobacteria* | *Caulobacterales* | *Caulobacteraceae* | *Phenylobacterium* | *Phenylobacterium_sp._1.9217* | |
| HQ014631 | Lake | 0.48 | 0.027 | 10 | *Bacteroidetes* | *Sphingobacteriia* | *Sphingobacteriales* | *Saprospiraceae* | *uncultured* | *uncultured_bacterium* | |
| KC502954 | Lake | 0.47 | 0.042 | 3 | *Proteobacteria* | *Gammaproteobacteria* | *Pseudomonadales* | *Pseudomonadaceae* | *Pseudomonas* | *uncultured_Pseudomonas_sp.* | |
