# Supplementary material for: Spatial heterogeneity and hydrological fluctuations drive bacterioplankton community composition in an Amazon floodplain system
Source: PLoS One. 2019 Aug 9;14(8):e0220695. doi: 10.1371/journal.pone.0220695 (PMC6688838; doi:10.1371/journal.pone.0220695)
Supplement: S1 Fig — (PDF) [file pone.0220695.s001.pdf]

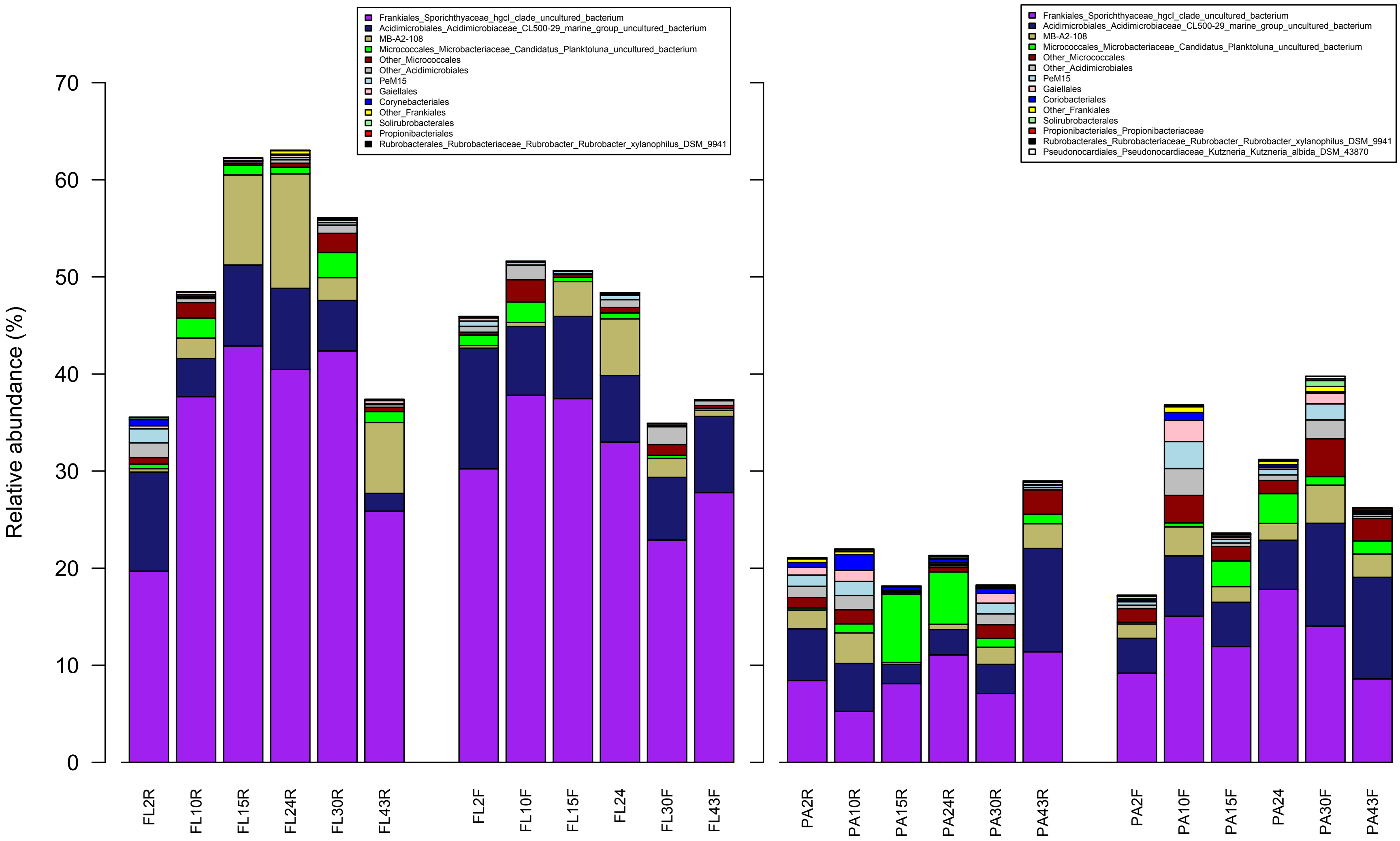

Frankiales_Sporichthyaceae_hgcl_clade_uncultured_bacterium
Acidimicrobiales_Acidimicrobiaceae_CL500-29_marine_group_uncultured_bacterium
MB-A2-108
Micrococcales_Microbacteriaceae_Candidatus_Planktoluna_uncultured_bacterium
Other_Micrococcales
Other_Acidimicrobiales
PeM15
Gaiellales
Corynebacteriales
Other_Frankiales
Solirubrobacterales
Propionibacteriales
Rubrobacterales_Rubrobacteriaceae_Rubrobacter_Rubrobacter_xylanophilus_DSM_9941
Frankiales_Sporichthyaceae_hgcl_clade_uncultured_bacterium
Acidimicrobiales_Acidimicrobiaceae_CL500-29_marine_group_uncultured_bacterium
MB-A2-108
Micrococcales_Microbacteriaceae_Candidatus_Planktoluna_uncultured_bacterium
Other_Micrococcales
Other_Acidimicrobiales
PeM15
Gaiellales
Coriobacteriales
Other_Frankiales
Solirubrobacterales
Propionibacteriales_Propionibacteriaceae
Rubrobacterales_Rubrobacteriaceae_Rubrobacter_Rubrobacter_xylanophilus_DSM_9941
Pseudonocardiales_Pseudonocardiaceae_Kutzneria_Kutzneria_albida_DSM_43870
Relative abundance (%)
0
10
20
30
40
50
60
70
FL2R
FL10R
FL15R
FL24R
FL30R
FL43R
FL2F
FL10F
FL15F
FL24
FL30F
FL43F
PA2R
PA10R
PA15R
PA24R
PA30R
PA43R
PA2F
PA10F
PA15F
PA24
PA30F
PA43F
